# Agricultural land use shapes dispersal in white-tailed deer (*Odocoileus virginianus*)

**DOI:** 10.1186/s40462-022-00342-5

**Published:** 2022-10-26

**Authors:** Marie L. J. Gilbertson, Alison C. Ketz, Matthew Hunsaker, Dana Jarosinski, Wesley Ellarson, Daniel P. Walsh, Daniel J. Storm, Wendy C. Turner

**Affiliations:** 1 Wisconsin Cooperative Wildlife Research Unit, Department of Forest and Wildlife Ecology, University of Wisconsin–Madison, 1630 Linden Dr, 53706 Madison, WI USA; 2 Wisconsin Department of Natural Resources, 1500 N Johns St, 53533 Dodgeville, WI USA; 3 Warnell School of Forestry and Natural Resources, University of Georgia, 180 E Green St, 30602 Athens, GA USA; 4 U.S. Geological Survey, Montana Cooperative Wildlife Research Unit, University of Montana, 32 Campus Drive NS 205, 59812 Missoula, MT USA; 5 Wisconsin Department of Natural Resources, 1300 West Clairemont Ave, 54701 Eau Claire, WI USA; 6 U.S. Geological Survey, Wisconsin Cooperative Wildlife Research Unit, Department of Forest and Wildlife Ecology, University of Wisconsin-Madison, 1630 Linden Dr, 53706 Madison, WI USA

**Keywords:** Cervid, Chronic wasting disease, Agricultural land use, Gene flow, Movement barriers, Pathogen spread, Resource selection, Social ecology, Step selection analysis, Wisconsin

## Abstract

**Background:**

Dispersal is a fundamental process to animal population dynamics and gene flow. In white-tailed deer (WTD; *Odocoileus virginianus*), dispersal also presents an increasingly relevant risk for the spread of infectious diseases. Across their wide range, WTD dispersal is believed to be driven by a suite of landscape and host behavioral factors, but these can vary by region, season, and sex. Our objectives were to (1) identify dispersal events in Wisconsin WTD and determine drivers of dispersal rates and distances, and (2) determine how landscape features (e.g., rivers, roads) structure deer dispersal paths.

**Methods:**

We developed an algorithmic approach to detect dispersal events from GPS collar data for 590 juvenile, yearling, and adult WTD. We used statistical models to identify host and landscape drivers of dispersal rates and distances, including the role of agricultural land use, the traversability of the landscape, and potential interactions between deer. We then performed a step selection analysis to determine how landscape features such as agricultural land use, elevation, rivers, and roads affected deer dispersal paths.

**Results:**

Dispersal predominantly occurred in juvenile males, of which 64.2% dispersed, with dispersal events uncommon in other sex and age classes. Juvenile male dispersal probability was positively associated with the proportion of the natal range that was classified as agricultural land use, but only during the spring. Dispersal distances were typically short (median 5.77 km, range: 1.3–68.3 km), especially in the fall. Further, dispersal distances were positively associated with agricultural land use in potential dispersal paths but negatively associated with the number of proximate deer in the natal range. Lastly, we found that, during dispersal, juvenile males typically avoided agricultural land use but selected for areas near rivers and streams.

**Conclusion:**

Land use—particularly agricultural—was a key driver of dispersal rates, distances, and paths in Wisconsin WTD. In addition, our results support the importance of deer social environments in shaping dispersal behavior. Our findings reinforce knowledge of dispersal ecology in WTD and how landscape factors—including major rivers, roads, and land-use patterns—structure host gene flow and potential pathogen transmission.

**Supplementary information:**

The online version contains supplementary material available at 10.1186/s40462-022-00342-5.

## Background

Wildlife dispersal—the act of permanently moving from what is typically a natal range to an adult range—is a key biological process which affects population dynamics and gene flow [1–3], and can contribute to the geographic spread of pathogens [4–6]. Dispersal behavior is generally thought to be motivated by inbreeding avoidance and intraspecific competition for mates or resources [3, 7–10]. Furthermore, when dispersing, landscape features may shape individual animals’ dispersal paths, thereby contributing to population connectivity [11, 12]. Determining how the landscape and an animal’s social environment shape dispersal behavior and movements is therefore critical for understanding wildlife biology and population dynamics.

White-tailed deer (*Odocoileus virginianus*; WTD or deer, hereafter), while heavily studied, are found across varied landscapes with a range of potential drivers for dispersal behaviors. For example, deer dispersal rates and distances increase in areas of low forest cover [13, 14] and high deer densities (e.g., [15], but see [13, 16]). Dispersal dynamics can also be temporally variable as in Clements et al. [17], where dispersal distance and direction varied by season. While deer dispersal is most common in juvenile males, some regions have seen higher rates of juvenile female dispersal (e.g., [14, 15, 18]). Drivers of dispersal vary by sex, with female deer dispersal thought to be density-dependent and motivated by access to resources [15], whereas male dispersal is driven by inbreeding avoidance and mate competition [7]. Furthermore, intersexual aggression from females towards juvenile male offspring increases spring dispersal probabilities, particularly with higher female deer densities [7, 19], while intrasexual aggression among males prior to the breeding season contributes to increased fall dispersal probabilities [7]. Once dispersal has been initiated, landscape features or barriers affect cervid movement paths. For example, major roads and rivers often act as semipermeable barriers (occasionally or rarely crossed) to movement [20–22] and subsequent gene flow (and likely pathogen spread; [23, 24]). Riparian areas appear to direct some dispersal movement [17], so while both rivers and roads may act as broad landscape barriers, rivers and streams could be particularly important for shaping the direction of travel. Forest cover is the most consistent driver of WTD dispersal distances [13], but the effect of landscape features on deer dispersal rates and distances is not always consistent across populations [22]. Bauder et al., [25], for example, found minimal landscape-driven genetic structuring of WTD in Ohio, concluding that landscape features likely do not form a barrier to deer movement in their study area.

Variability in dispersal patterns between sexes, temporal periods, and geographic regions results in uncertainty regarding movement ecology and population connectivity or gene flow across deer populations—uncertainty that has important consequences for deer population management. These consequences are particularly apparent in the context of disease spread. For example, chronic wasting disease (CWD) is a fatal prion disease of cervids, and is increasing in prevalence and geographic distribution across North America [26]. As CWD prevalence increases, the probability of juvenile infection prior to dispersal is also expected to increase [27, 28]. Thus, in addition to shaping population gene flow, juvenile dispersal events present an increasing risk for geographic disease spread. Understanding drivers of deer dispersal behavior and movements thereby informs deer population management and disease control and surveillance efforts [29, 30].

Our objectives were to (1) identify dispersal events in Wisconsin WTD and determine drivers of dispersal rates and distances, and (2) determine how landscape features (e.g., rivers, major roads) structure deer dispersal paths. We expected that juvenile males would complete most dispersal events, and predicted that land use—especially forested versus agricultural land use [13, 14]—and deer social environments [15] would be key drivers of deer dispersal behavior and movement trajectories. More specifically, we predicted that individuals in natal ranges with limited access to forest cover or in areas with an increased number of proximate individuals would be more likely to disperse [7, 13]. We expected that agricultural land use would favor longer dispersal distances as dispersing individuals seek out key cover [13]. Lastly, we predicted that landscape barriers like major rivers and roads would further shape dispersal patterns by acting as semipermeable barriers to dispersal [20, 22]. For example, if potential dispersal paths for an individual frequently intersected or intersected soon after origin with major roads or rivers, we expected the individual would be less likely to disperse (i.e., “frustrated dispersal”; [31, 32]).

## Methods

### Study area and deer collaring

The Wisconsin Department of Natural Resources (WDNR), in collaboration with > 300 landowners, captured 1,157 individual WTD from 2017 to 2020 in Iowa, Grant, and Dane counties in southwestern Wisconsin, as part of ongoing research on CWD. As the region in which CWD was first detected in Wisconsin in 2001 [33], our study area has featured in extensive prior research (e.g., [34–36]). The habitat in this area has a rolling topography with highly dissected deciduous forest patches (about 41.0% deciduous and 4.2% mixed or evergreen forest) interspersed with agricultural land use (about 19.9% pasture or hay and 21.8% cultivated crops) and minor amounts of grassland and woody or emergent herbaceous wetlands. Our study was rural, with several small towns, located directly west of the city of Madison, Wisconsin.

Deer were captured using a combination of clover traps [37], drop nets [38], box traps [39], and chemical immobilization with intramuscular injections of BAM (27.3 mg/ml butorphanol + 9.1 mg/ml azaperone + 10.9 mg/ml medetomidine; [40]) via hand-injection. During capture, deer were monitored via rectal temperature, respiratory and heart rates, and capillary refill time. Deer were subsequently chemically mobilized with atipamezole (25 mg/ml). Deer capture and handling protocols were approved under WDNR’s Animal Care and Use Committee (Protocol: 16-Storm-01).

At the time of capture, 763 deer (452 female, 311 male) greater than 8 months of age were fitted with global positioning system (GPS) collars (Vectronic VERTEX Lite Iridium or Lotek LiteTrack Iridium 420) and biological samples (incisiform tooth if estimated > 20 months of age) and measurements (body weight, tooth replacement and wear) were recorded. Captures occurred from December to March each year, and at capture, individuals were classified as *juvenile* (approximately 8 months of age), *yearling* (approximately 20 months of age), or *adult* (greater than two years of age) by body size and tooth wear [41], or incisor cementum annuli [42, 43] in cases where the former two measures were inconclusive. GPS collars recorded deer locations typically every four hours, but as often as hourly during fawning or dispersal seasons (i.e., spring and fall), or as infrequently as every 23 h to conserve battery life.

### GPS collar data processing

Deer GPS collar data were processed to screen for likely erroneous locations before analysis. For GPS collar data preparation, Lewis et al. [44] recommend removing 2D fixes above a dilution of precision (DOP) cutoff (in their case, DOP > 5), while Bjørneraas et al. [45] recommend using movement characteristics, such as “spike” or high-speed movements, to screen data for likely errant points. We used a hybrid of these two recommendations, removing 2D points with DOP > 5, and screening for spike movements. Spike movements were defined and removed based on having a turning angle between 166–194º and either (1) a high displacement rate, relative to the population (in the 99th percentile of displacement rates across the study population; 0.67 km/hr), or (2) crossing and immediately returning across the Wisconsin River. In addition, we trimmed the ends of all trajectories to account for capture or mortality effects on movement. For all individuals, we removed 3 days from the start of the animal’s trajectory, and 1 day from the end of the trajectory. This approach helped ensure data removals were both biologically relevant and consistent with the quality of the data.

Following processing, we included only those individuals with movement data spanning at least one *dispersal season*, including data for a season only if it included at least 60 recorded locations. For this screening process, we defined dispersal seasons as spring (1 Apr − 31 Jul) or fall (1 Sep − 31 Dec; these windows conservatively expand beyond peak dispersal seasons typically described as May-Jun and Sep-Nov; [13, 22]). We evaluated male and female deer across all age classes (juveniles, yearlings, and adults). We evaluated adults to screen for evidence of range shifts among older age classes, especially those consistent with migratory behavior which would affect our interpretation of dispersal behavior (i.e., migratory behavior could be mis-classified as dispersal events).

### Dispersal detection algorithm

Following Lutz et al. [15, 19], we defined dispersal as a permanent, one–way movement from a natal range (hereafter, *pre-dispersal range*) to a new range (*post-dispersal range*). Further, to be defined as a dispersal event, the individual’s post-dispersal range could not overlap with the pre-dispersal range. Based on this definition, we developed an automated algorithm for identification of dispersal to facilitate the analysis of a large number of individuals.

For each deer, we used *k*-means clustering to identify geographical *clusters* of locations [46]. We used the silhouette approach [47, 48] to determine the number of clusters to test per individual, limiting the algorithm to testing for 2–5 clusters. Most individuals for which *k*-means identified more than 2 clusters were non-dispersing individuals, so increasing the number of clusters was unlikely to improve dispersal detection.

After cluster detection, we then determined the spatial overlap between the identified clusters. If a cluster contained at least 30 locations, we quantified overlap using the utilization distribution overlap index (UDOI) of the 95% vertices of bivariate normal home range kernels [49]. For efficiency, we used the default settings in the R package *adehabitatHR* [50]. If a cluster contained fewer than 30 locations, we determined if any of the cluster’s points fell within the 95% kernel density estimation (KDE) home range vertices of other clusters. We note that KDE approaches can underestimate home range size when movement data are autocorrelated [51], but autocorrelated KDE (aKDE) approaches can be prohibitively slow for large datasets such as ours. We found, however, that home range sizes derived for a sample of our data using KDE versus aKDE approaches were not significantly different (supplementary methods), so our dispersal detections are unlikely to have been affected by this choice.

We used the cluster overlap results to classify each individual as a *disperser* or *non-disperser*. Dispersers were classed as those individuals for which (1) at least one cluster did not overlap with any other clusters, and (2) the last-used cluster was different from and not overlapping the first-used cluster. All other individuals were considered non-dispersers (see Figure S1 for examples of the clustering and classification process). Dispersal classifications for all individuals were visually screened and updated (*reclassified*) if the automated workflow resulted in an apparent misclassification.

### Descriptive analysis

We defined dispersal distance as the Euclidean distance between pre- and post-dispersal cluster centers (as determined by *k*-means above). We similarly defined the dispersal direction as that from the pre- to post-range cluster center, and tested for bias in dispersal direction across dispersers using CircMLE [52, 53]. CircMLE determines directional bias using a model selection approach across the 10 models of orientation described by Schnute and Groot [54], with the uniform model (i.e., no directional bias) as the null model. Output of CircMLE gives the top orientation model, which can include up to two directional distributions if directional bias is not unimodal; for each directional distribution, CircMLE provides the mean direction and concentration parameters.

Among dispersers, we defined the duration and timing of dispersal as the time from the last GPS location within the pre-dispersal range to the first GPS location within the post-dispersal range. These ranges were defined as the vertices for the 95% KDE home range from previous *k*-means analysis. Because GPS locations could fall outside the 95% KDE vertices on a number of occasions besides the main dispersal event, for all dispersing individuals we defined a preliminary “dispersal window” based on visual inspection of an individual’s net-squared displacement (similar to the approach used by [21]). Within this window, we then identified the last location in the pre-dispersal range and the first location in the post-dispersal range. As with dispersal classification, all timing estimations were visually inspected for accuracy (see supplementary materials for more details, and Figure S2 for examples of the dispersal timing estimation process).

### Statistical models

Our two response variables were dispersal (a binary outcome) and log-transformed dispersal distance (in km). While our descriptive analysis (above) examined individuals of all age and sex classes, for the statistical models for dispersal and dispersal distance, we only evaluated juvenile males due to limited dispersal events in other age and sex classes. Further, we only evaluated individuals with pre-dispersal collar data spanning at least 40 days to ensure accurate covariate estimation. Because many non-dispersers were observed in both spring and fall, we ran separate spring and fall models to account for potentially different seasonal drivers. All dispersing and non-dispersing individuals had a pre-dispersal range, which we defined based on all GPS locations from 1 Mar or 1 Aug to either the date of dispersal (for dispersers) or the median date of dispersal for a given season (for non-dispersers; spring: 25 May; fall: 22 Oct). We evaluated dispersal distance for dispersers only and did not run separate seasonal models. We predicted that dispersal probability and distance for juvenile male deer would each be a function of *access to resources* (i.e., cover, proximate individuals) and the *ability to disperse* (i.e., traversability of the surrounding landscape, individual condition), as reflected by our model covariates described below. A table of relevant data sources used in all analyses is available in the supplementary materials (Table S1). All analyses were performed in R v3.6.3 and 4.1.2 [55].

#### Access to cover

To test for a relationship between access to cover and dispersal probability and distance, we used National Land Cover Database (NLCD, 30 m resolution; [56]) designations to calculate the proportion of each individual’s pre-dispersal range that was classified as “planted or cultivated” (the NLCD planted/cultivated designations include pasture/hay and cultivated crops, which we hereafter refer to as *proportion agricultural*) or forested (deciduous, evergreen, or mixed forest, though our study region was classified almost exclusively as deciduous forest). For estimating these proportions, we used the 95% aKDE home range vertices to define range borders (supplementary materials), and removed home range size outliers (greater than three standard deviations larger than the mean; n = 3). We used aKDE home ranges for this analysis because the smaller sample sizes here, as compared to our dispersal detection algorithm, mitigated the computational demands of aKDE approaches, and because we felt this habitat analysis would most benefit from reducing the risk of bias or error in home range estimation introduced by autocorrelated movement data [51, 57]. Because the proportion agricultural was strongly negatively correlated with the proportion forested (Pearson correlation coefficient = -0.76), our statistical analyses incorporated only the proportion agricultural.

#### Social environment

We lacked fine-scale or sex-specific deer density data, so we quantified the deer social environment with two metrics, hereafter *average proximity* and *number proximate*. For average proximity, we first calculated *proximity scores* between all pairs of collared individuals. Proximity score was defined as the number of GPS locations within a 50 m distance threshold per all simultaneous locations (with a 60 min allowance) between a given pair of GPS collared individuals [58, 59]. For each juvenile male, we then averaged its pairwise proximity scores across all *proximate individuals* (those with a proximity score greater than zero) to generate its average proximity value. Proximity scores were likely to be biased for individuals with few proximate individuals; we therefore only evaluated those individuals with an average proximity score greater than zero and with at least five collared individuals “available” (having GPS locations) within 4 km of that individual’s range center. For the number proximate metric, we calculated the number of unique proximate individuals for each juvenile male, relative to the number of available individuals of any sex or age within 4 km of the focal individual’s range center.

The average proximity metric represents the relative frequency with which juvenile males “associated” with their potential contacts, and the number of proximate individuals represents the number of unique individuals “associated with,” relative to the number of available individuals nearby. We emphasize that these “associations” do not necessarily represent direct interactions, but are meant to represent events where an individual could reasonably detect the recent presence of a conspecific. While collaring effort—especially geographically biased collar deployment—may bias social interaction metrics, social network studies have shown that those metrics which quantify the number and relative frequency of interactions appear to be the most robust to undersampling [60–62], particularly among clustered or highly social species like deer [62]. However, to determine robustness of our results, we performed an additional sensitivity analysis for the effect of collaring effort on the number proximate metric (supplementary materials).

#### Traversability of surrounding landscape

To quantify the traversability of the landscape surrounding each individual, we simulated potential dispersal paths. We attempted to fit hidden Markov models (HMM) with two or three behavioral states to each dispersing individual’s movement trajectory (regularized to fixes every four hours using continuous time movement modeling; [63, 64]). Dispersal events were often short in both duration and distance, but HMMs identified a movement state consistent with dispersal for 24 individuals. We averaged the movement parameters for this dispersal movement state and used them to simulate 100 movement trajectories per individual. Each simulated path initiated at a randomly selected used location within the focal individual’s pre-dispersal range. To align with the average dispersal duration, simulated movements were for 11 steps, with four hours per step.

With the simulated trajectories, we calculated (1) the mean first step in poor habitat (defined as “developed” or “water” by NLCD) across the 100 simulations, (2) the mean proportion of steps per simulated path that were in agricultural land use, (3) the proportion of simulated paths that intersected with major roads, and (4) the proportion of simulated paths that intersected with rivers or streams (Table S1). The traversability agricultural land use metric was only used in the dispersal distance model, and, being highly correlated with the proportion of agricultural land use in pre-dispersal ranges (Pearson correlation coefficient = 0.73), these two variables were never included in models together. Our simulated paths approach allowed us to quantify traversability without calculating a semi-arbitrary dispersal buffer for extracting landscape covariates in the surrounding area. In addition, our approach allowed dispersal path extents to derive from and therefore correspond to the focal individual’s space use (i.e., did not assume circular home ranges). Because simulated paths reached variable Euclidean distances from the initial range, habitat closer to the initial range was more likely to contribute to traversability metrics. Given that dispersal theory suggests that individuals disperse to the first available range and then stop to reduce risk [3, 7], we believe the emergent weighting of closer habitat values was biologically reasonable.

#### Individual condition

We used body weight as a proxy for deer body condition, expecting that deer in better condition would be more able to disperse and disperse longer distances than those in poorer body condition. Deer body weight was recorded at capture; because captures typically occurred between December and March each year, capture weights were expected to be more informative for spring dispersals.

#### Statistical model fitting

For both response variables, dispersal and dispersal distance, we fit models based on specific biological hypotheses [65]. Dispersal models were logistic regressions, while models for log-transformed dispersal distances were linear regressions (Table 1). For the logistic regression of dispersal events, we had several “null” hypothesis models that evaluated dispersal as a function of (1) aKDE-based home range area, (2) number of pre-dispersal fixes, (3) longitude of capture location, or (4) year. The home range area null model tested if dispersal was simply a function of home range size (e.g., dispersal more likely with small home ranges). The null model for the number of pre-dispersal fixes tested for the possibility of increased dispersal detection with increased GPS locations and was not biologically motivated but controlled for improved identification of range shifts with increased location data. The longitude of capture location was included in the null modeling to account for the possibility of an unmeasured factor correlated with the slight east-west gradient in agricultural land use in our study area. The null model for year tested for variability in dispersal rates across the years of our study; because stratifying by year limited sample sizes on a per-year basis (minimum of three juvenile male dispersers in one year), we chose to exclude a year effect in full models. The null models for log-dispersal distance (linear regression) were identical to those for dispersal probability, with the addition of a null model for season that tested for the role of season alone in describing dispersal distances, and motivated our choice to include season as a covariate in all full models testing our broader hypotheses (Table 1).

Not all individuals had data for proximity metrics or body weight, so we fit separate full hypothesis models by data availability to maximize data use and confirm consistency of findings. We fit three classes of full models representing our major hypotheses: (1) models with maximum individuals, but without proximity or body weight covariates; (2) models with the addition of one proximity metric (used to determine top proximity metric via Akaike information criterion, AIC; [65, 66]), and (3) models with the fewest individuals, but including all covariates. All predictors were scaled and centered, and models were checked graphically for linearity assumptions (binned residuals for logistic regressions, residuals for linear regressions; [67, 68]).

**Table 1:** Summary of statistical models evaluating Wisconsin white-tailed deer dispersal

Response	Hypothesis class	Covariates
Dispersal* (logistic regression)	Null	aKDE-based home range area
		Number of pre-dispersal fixes
		Longitude of capture location
		Year
	Full	Agricultural + average first poor location + proportion potential paths intersecting roads
		Agricultural + average first poor location + proportion potential paths intersecting roads + average proximity OR number proximate
		Agricultural + average first poor location + proportion potential paths intersecting roads + number proximate + body weight
Log-transformed dispersal distance (linear regression)	Null	aKDE-based home range area
		Number of pre-dispersal fixes
		Longitude of capture location
		Year
		Season^†^
	Full	Proportion potential paths intersecting rivers and streams + proportion intersecting roads + season*average proportion of steps falling in agricultural land OR season*agricultural
		Proportion potential paths intersecting rivers and streams + proportion intersecting roads + season*average proportion of steps falling in agricultural land + season*average proximity OR season*number proximate
		Proportion potential paths intersecting rivers and streams + proportion intersecting roads + season*average proportion of steps falling in agricultural land + season*number proximate + body weight

Note: Models with “OR” indicate variables that were not included in models together, but were selected via AIC. *Dispersal logistic regressions were fit separately for spring and fall models. ^*†*^Season was highly significant for dispersal distances and was therefore included in all dispersal distance models. Abbreviated covariates were: aKDE home range area = autocorrelated kernel density estimation home range area; agricultural = proportion of pre-dispersal range classified as planted or agricultural land use; average first poor location = the average first simulated dispersal step in “developed” or “water” land types; proportion intersecting roads = proportion simulated paths intersecting roads; average proximity = an individual’s average proximity score across potential associations; number proximate = the number of individuals proximate to the focal individual (per available individuals within 4 km). Season was spring or fall; Year was a categorical variable for the years 2017–2020

### Dispersal habitat selection

To determine how landscape structures dispersal movements, we performed a step selection analysis for juvenile male deer. Step selection functions (SSFs) evaluate habitat covariates at consecutive versus matched available steps [69]. An extension of SSFs, integrated SSFs (iSSFs), include movement covariates and allow habitat selection to be jointly estimated [70–72]. We fit separate iSSFs for three different movement states: *pre-dispersal* movements (trajectories of dispersers prior to dispersal), *dispersal* movements, and *non-dispersal* movements (trajectories of non-dispersers prior to median date of dispersal). Pre-dispersal movements were included to act as “within-individual” controls, while non-dispersal movements could account for potential differences in habitat selection between dispersing and non-dispersing individuals prior to or during dispersal. For all movement states, we used trajectories with locations recorded every four hours. Because the frequency of location recording can affect iSSF results [69], for dispersal movements, we fit an additional iSSF with locations recorded every hour (see supplementary materials for additional iSSF details).

We fit iSSFs both at the *individual level* and at the *population level*. For individual-level models, we fit iSSFs for each individual separately using the *amt* package in R [72]. For the population-level models, because failure to account for random coefficients can lead to biased inference [73], we used a two-step estimation approach for mixed-effects models, including random coefficients for all covariates [74, 75] using the *TwoStepCLogit* package in R [76]. The two-step estimation approach frequently fails when individuals do not have enough covariate variability (e.g., those that do not encounter all levels of categorical predictors; [74]). As such, population-level models included only those individuals that had adequate covariate variability to fit individual-level models (pre-dispersal: n = 75; dispersal: n = 33 for locations every four hours, n = 38 for locations every one hour; non-dispersal: n = 45).

For all iSSFs, we used 16 random steps for each observed step, and performed a sensitivity analysis for this choice to confirm stability of resulting coefficients (Figure S3). Random step lengths and turning angles were based on independent gamma and von Mises distributions, respectively [72]. All iSSFs evaluated habitat covariates of agricultural land use (binary variable from NLCD), elevation (as a second order polynomial), and distance to the nearest river or stream (see Table S1 for data sources). In addition, we evaluated a binary “intersection with roads” covariate, which documented if real or random steps intersected with major roads. Road crossings were ultimately rare (dispersal movements: 2.5% real and 4.7% of random steps intersected roads; pre-dispersal: 0.02% of real and 0.18% of random; non-dispersal: 0.03% of real and 0.18% of random), so we only used this covariate in an additional, separate model with the subset of individuals with dispersal movements that included road crossings (n = 14).

All iSSFs also included the movement-related covariates of step length, log-step length, and cosine of turning angles. We expected that deer would exhibit faster and more directed movement (i.e., longer step lengths, more concentrated turning angle distributions) when in low-cover, agricultural environments. We therefore included interactions between agricultural land use and movement covariates. All main effects habitat covariates were extracted at the end of steps, and interaction coefficients at the start of steps (see Table 2 for full iSSF model specifications). Log-relative selection strength plots were generated using coefficient estimates and average covariate values [77, 78].

**Table 2:** Step selection analysis terms and descriptions for Wisconsin white-tailed deer

Term	Description or prediction
Step length	Estimator for scale parameter of gamma step-length distribution
log(Step length)	Estimator for shape parameter of gamma step-length distribution
cosine(Turning angle)	Estimator for concentration parameter of von Mises turning angle distribution
Agricultural (end of step)	Avoidance of agricultural land use during dispersal movements
Elevation + Elevation^2^	Selection for intermediate elevation
Distance to rivers/streams	Avoidance of greater distance from nearest river/stream
Intersection with roads	Avoidance of road crossings
Agricultural (start): Step length	More variable step lengths when steps initiate in agricultural land use
Agricultural (start): log(Step length)	Longer step lengths when steps initiate in agricultural land use
Agricultural (start): cosine(Turning angle)	More concentrated turning angles (i.e., less variation from moving straight ahead) when steps initiate in agricultural land use

*Note: Model terms were included in all integrated step selection function models (i.e., dispersal, pre-dispersal, and non-dispersal movement iSSFs).*

## Results

**Fig. 1:**
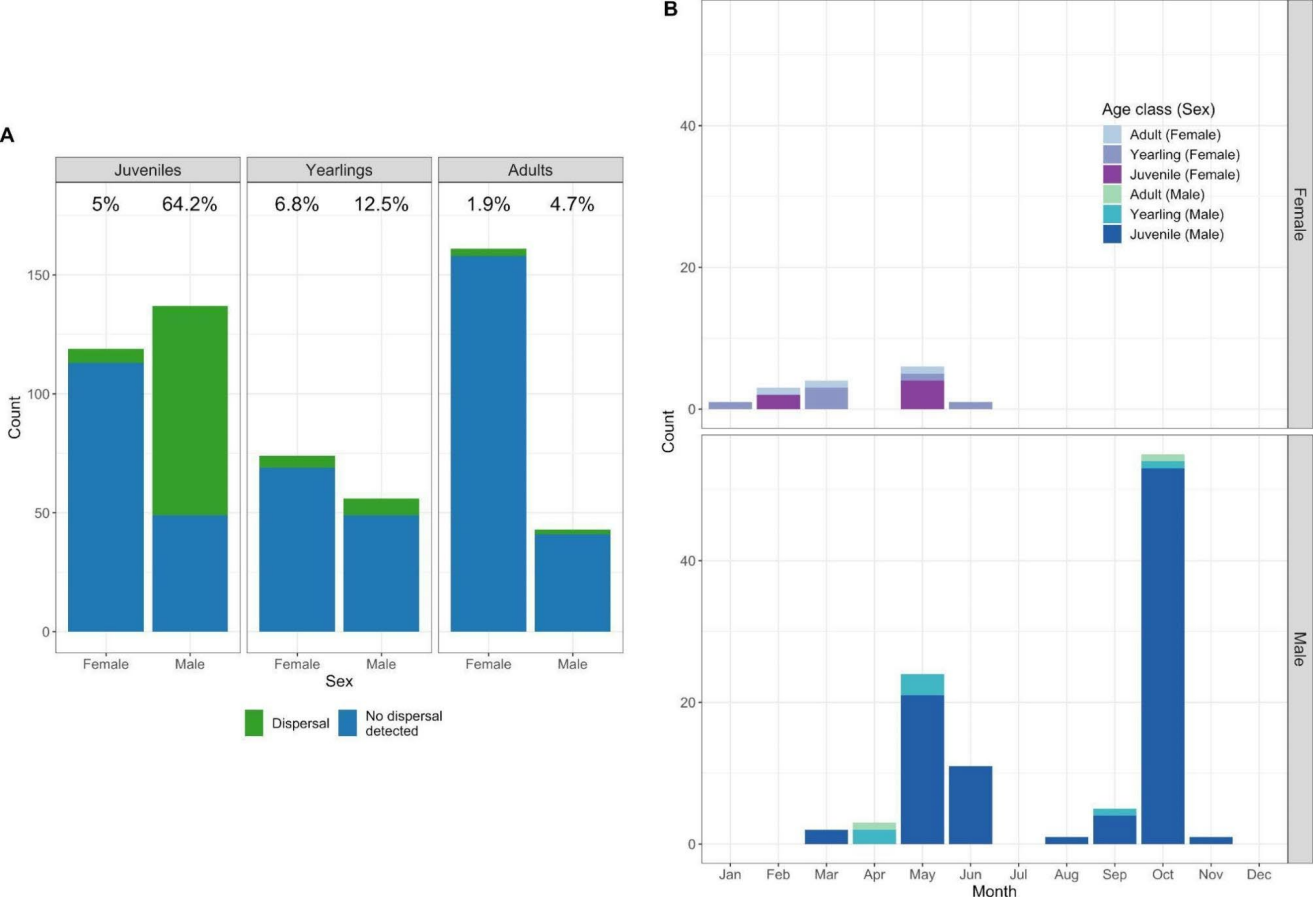
Counts of southwest Wisconsin white-tailed deer dispersal by sex and age class. In (A), dispersals are shown in green (top of bar) and percentages above each bar give the percent dispersers for each sex/age class combination. In (B), the timing of dispersals is shown by sex (females in upper panel, males in lower) and age class (color or shading of bars). Note that in (B), individuals that dispersed two times are shown twice to show the full distribution of dispersal events, and the y-axes are identical to facilitate visual comparison by sex

We evaluated 590 individual juvenile, yearling, and adult deer, of which we identified 111 individuals as dispersers (18.8%; see Table S2 for sample sizes across dispersal status, sex, and age class) after accounting for dispersal classification errors identified by visual inspection. Such errors were rare: 2.7% of analyzed individuals required reclassification, with the majority of these (87.5% of reclassifications) being changes from an initial dispersal classification to non-dispersal. Dispersal was most common in juvenile males, with 64.2% of these individuals classified as dispersers. Dispersal was uncommon for all other age and sex classes (Fig. 1). Six dispersers (five juvenile males and one yearling female) completed two distinct dispersal events, with the first event in the spring, and the second in the following fall (except for the yearling female, whose second shift occurred in January).

**Fig. 2:**
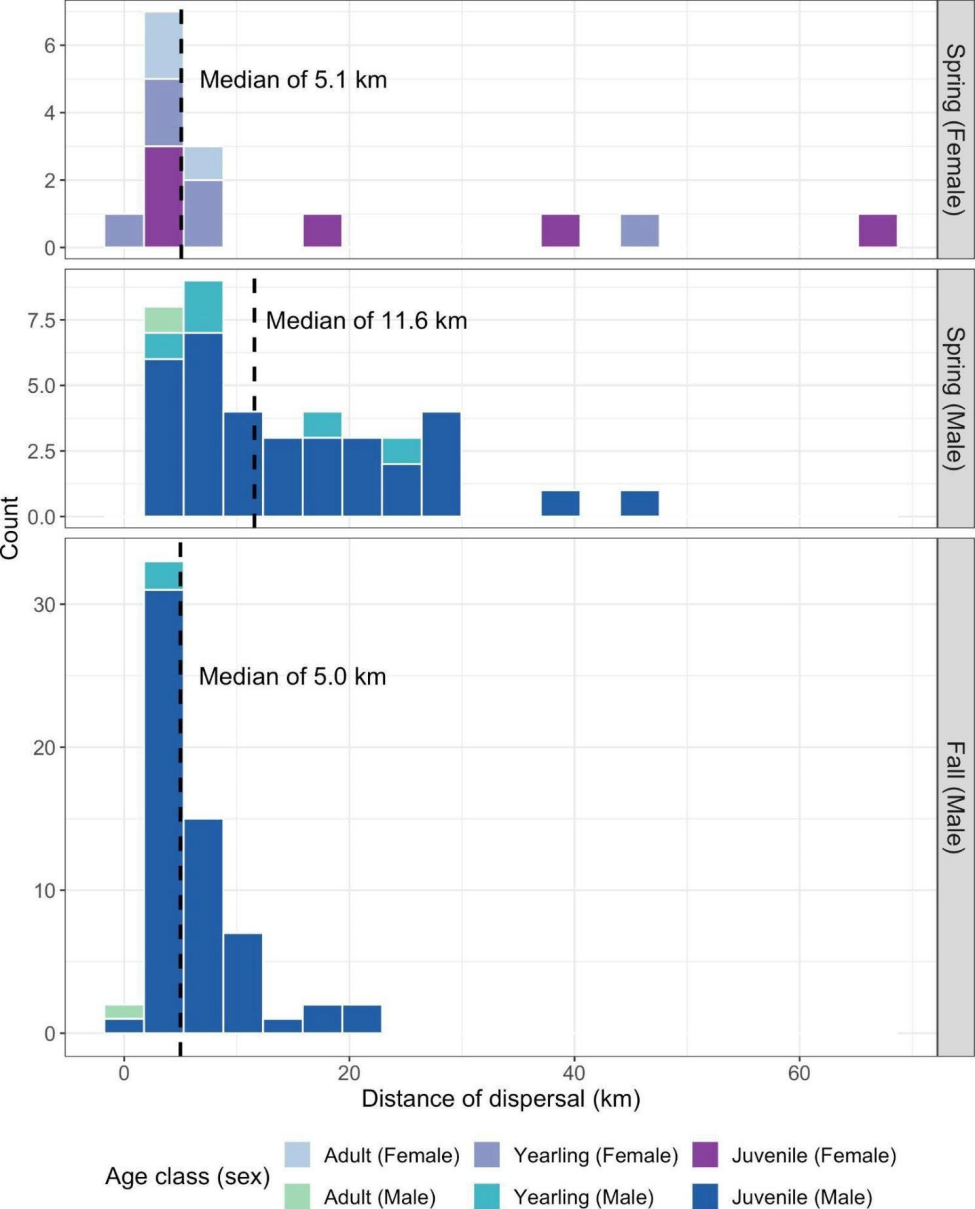
Histograms of Wisconsin white-tailed deer dispersal distances (in km), stratified by season and sex. Bars are colored by the age class of individuals at dispersal. Females only dispersed in the spring, and are shown in the top panel; male dispersal distances in the spring and fall are shown in the middle and bottom panels, respectively. Y-axes vary between panels, with the bottom panel the largest due to its higher count values. Median dispersal distances per season and sex are shown with vertical dashed lines. Note that individuals that dispersed two times (n = 6) are shown twice to show the full distribution of dispersal distances

We observed distinct spring and fall dispersal periods. Only males dispersed in the fall dispersal season, and yearling and adult dispersal (or range shift) events predominantly occurred in the spring (Fig. 1). We did not observe annual range shift patterns consistent with migratory movements. The median dispersal distance across all dispersers was 5.77 km (range: 1.3–68.3 km), and the median dispersal duration was 1.71 days (range: 1 h − 47.7 days). Dispersal distances and durations were typically shorter in the fall, even accounting for the lack of female dispersal in the fall (Fig. 2, S4). The longest distance dispersal events were completed by juvenile and sometimes yearling individuals (Fig. 1).

**Fig. 3:**
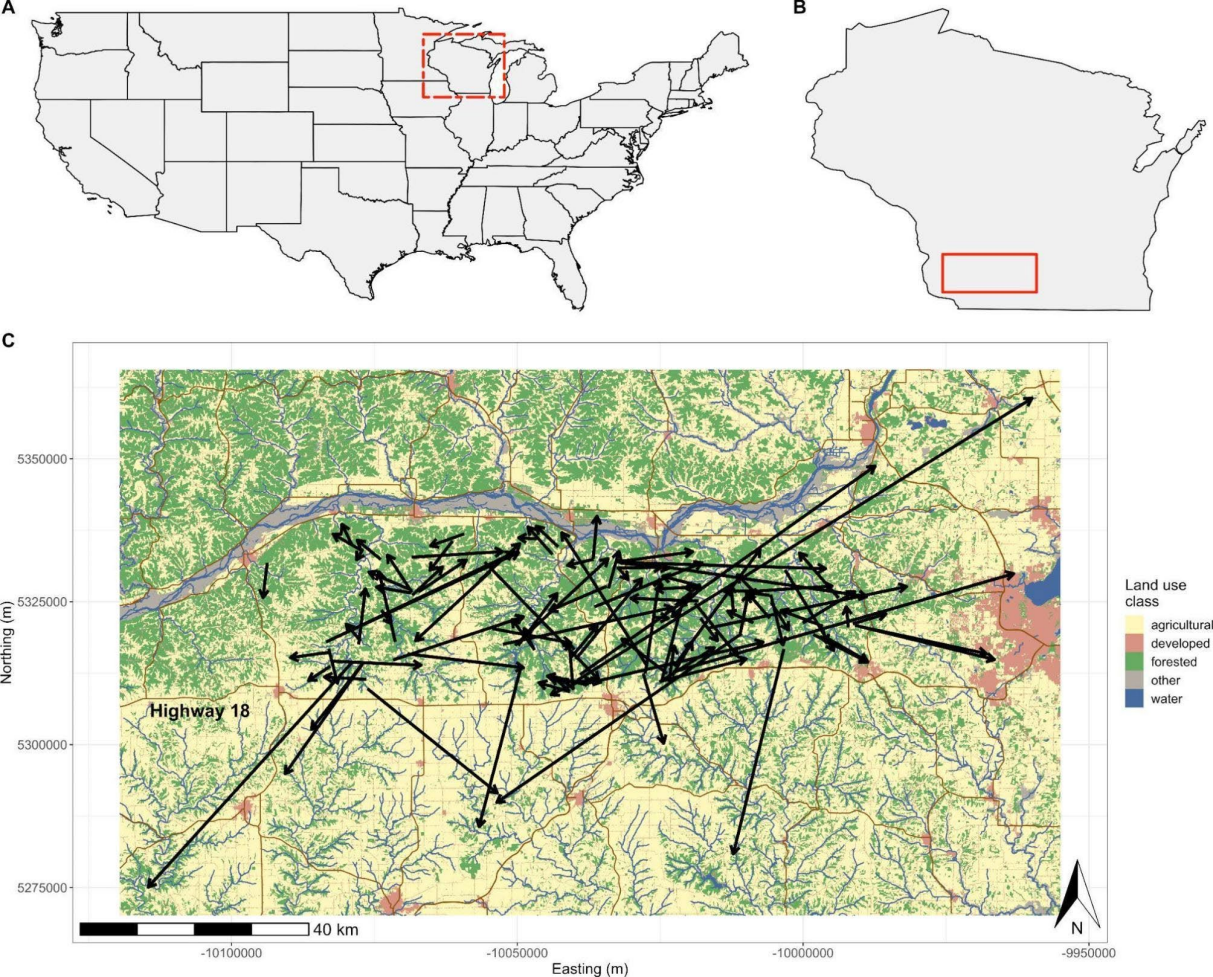
Maps showing (A) the study area locale of Wisconsin (red dashed box) in the context of the United States, (B) the study area and surrounding land (red solid box) in the context of Wisconsin, and (C) white-tailed deer dispersal events with the landscape colored by land use class. In (B), the solid red box corresponds to the bounds of the area shown in (C). The arrows in (C) initiate at each individual’s pre-dispersal range center and end at their post-dispersal range center. The dispersals for individuals that completed multiple dispersal events are shown as a single arrow connecting their first range and final range (i.e., each arrow represents a different dispersing individual). Map colors correspond to NLCD land use classifications; of the 4.4% of land pictured classified as “other,” about 84.3% is woody or emergent herbaceous wetlands, with the remainder a mix of barren land, shrub/scrub, and grassland/herbaceous. The label for Highway 18 indicates the major east-west highway that formed the southern boundary of our deer capture area. The red land use to the right is part of the urban area of Madison, WI; the major river pictured is the Wisconsin River

We observed an overall bias in dispersal directions towards the east and west-northwest (Fig. 3, S5, Table S3). When subsetting dispersals by season, models showed some support for seasonal variation in dispersal direction, with spring dispersals biased toward the east and fall dispersal directions bimodally distributed with a stronger west-northwest bias (Figure S5, Table S3).

### Dispersal probability

**Fig. 4:**
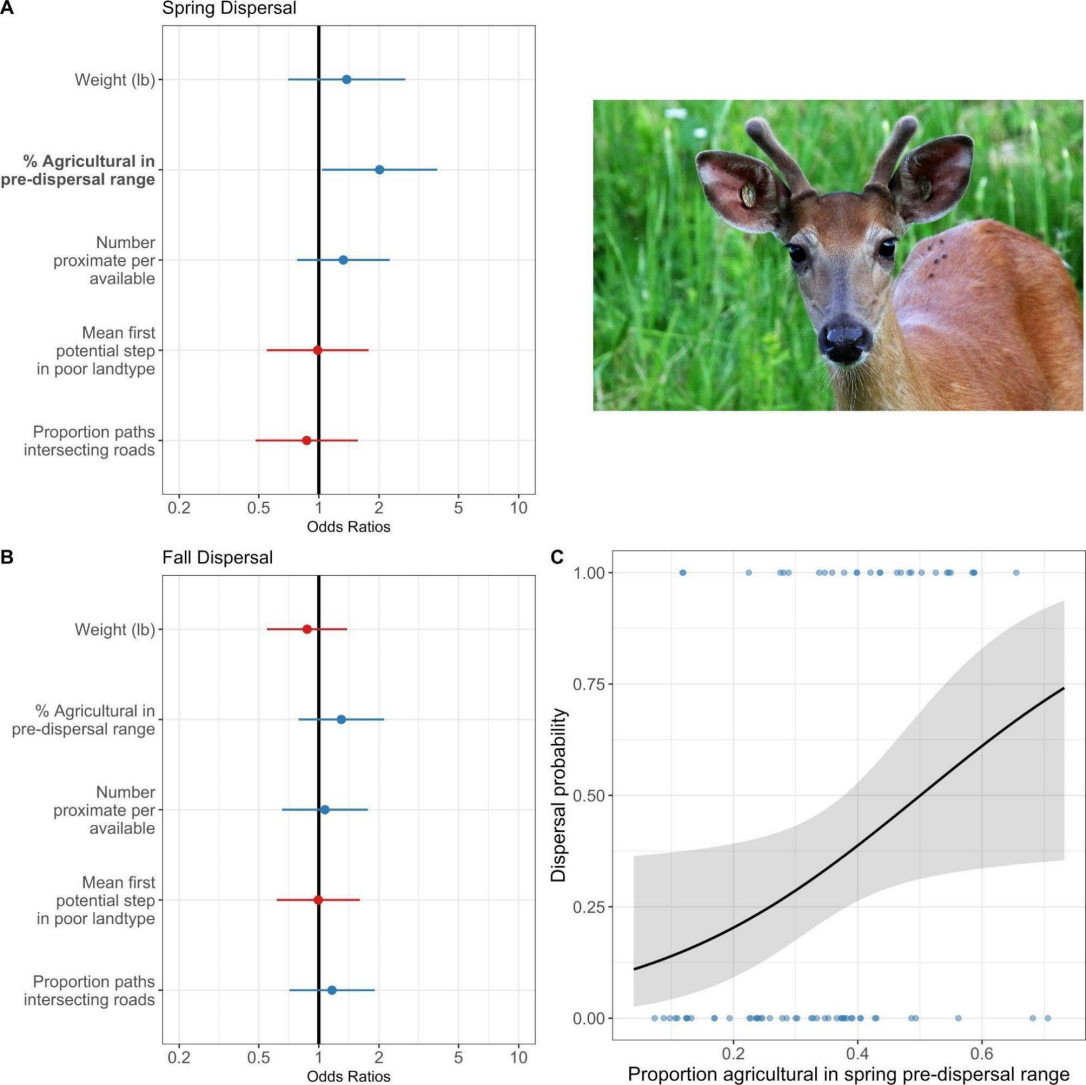
Dispersal logistic regression model results for juvenile male white-tailed deer in southwestern Wisconsin, including model estimates and confidence intervals in (A) spring and (B) fall, as well as (C) the effect of agricultural land use on dispersal probability in spring. For (A) and (B), blue results show positive coefficient estimates, red show negative coefficient estimates. Covariates were statistically significant (shown in bold text) if the 95% confidence interval did not cross the vertical black line (odds ratio = 1). For (C), the black line and gray ribbon show the effects estimate and 95% confidence interval, respectively, from the spring model in (A). Blue dots in (C) are data points. All predictors were scaled and centered. Deer photo credit to Jerry Davis

Our logistic regression for juvenile male dispersal identified a seasonal effect of agricultural land use on dispersal probability (Fig. 4), with juvenile males having higher odds of dispersing with a higher proportion of agricultural land use in their pre-dispersal range, but only in the spring (Table S4). Based on null models, there was some evidence that detection of dispersals increased with the number of locations recorded in the spring (Table S5), such that dispersal rates among juvenile males may be higher in the spring than we detected here. Models did not identify clear drivers of dispersal in the fall (Fig. 4, Table S4), though there was no statistical difference in agricultural land use in pre-dispersal ranges among non-dispersers in the spring versus fall (two-sided paired t-test: *t* = -1.68, *p* = 0.10). Dispersal probability results were robust across all full models (Table S6).

### Dispersal distance

**Fig. 5:**
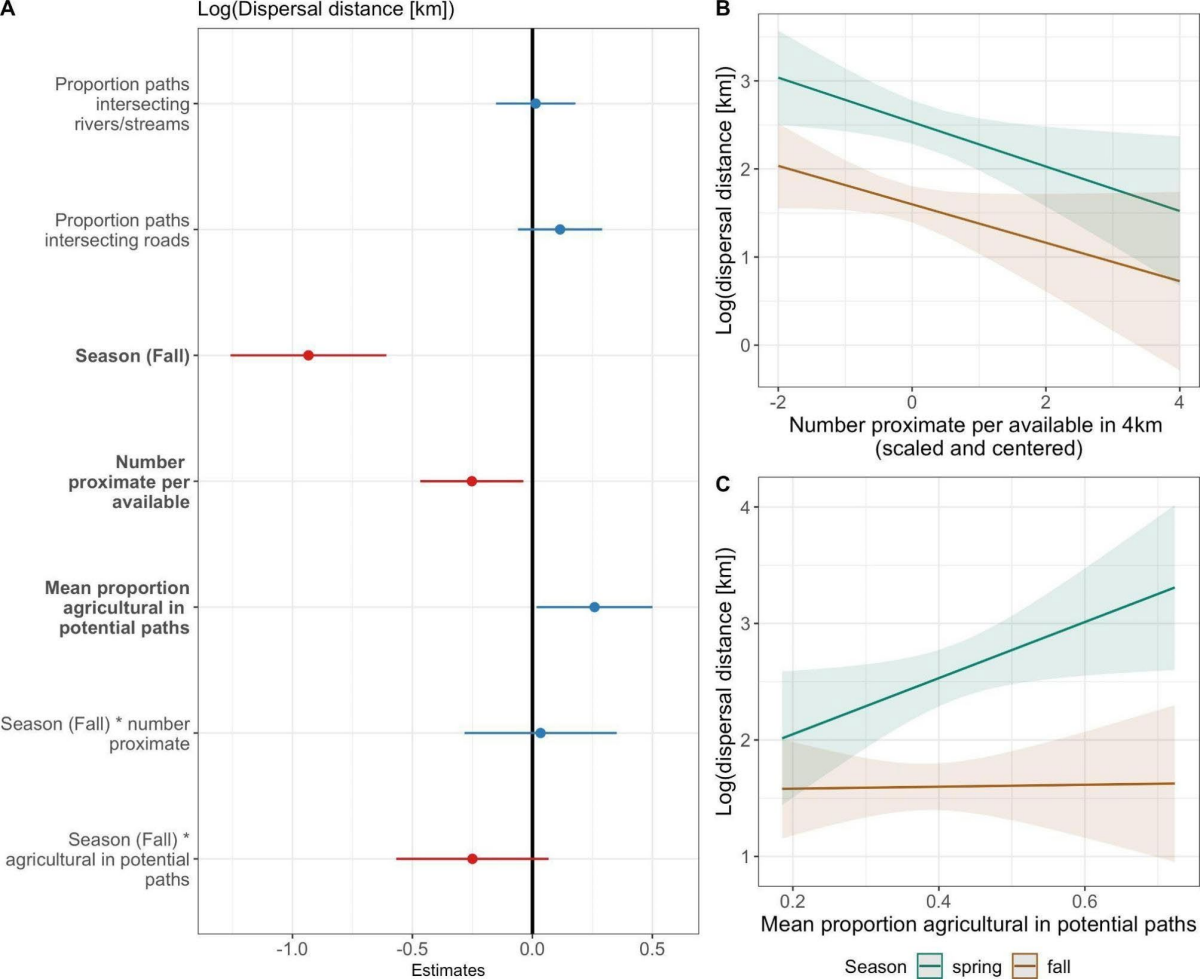
Model results for linear regression of log-transformed dispersal distance (not including body weight predictor) for juvenile male white-tailed deer in southwestern Wisconsin. Panels show (A) coefficient estimates and 95% confidence intervals, (B) the effect of the number of proximate individuals (per available) by season, and (C) the effect of agricultural land use in potential dispersal paths by season. In (A), blue results show positive coefficient estimates, red show negative coefficient estimates. Covariates were statistically significant (shown in bold text) if the 95% confidence interval did not cross the vertical black line (estimate = 0). For (B) and (C) spring effects estimates and 95% confidence intervals are shown in teal and fall in brown

Linear regression models for log-transformed dispersal distance among juvenile males identified a strong effect of season, with spring dispersals associated with longer dispersal distances than those in the fall (Fig. 5, Table S7). In addition, models identified an agricultural effect on dispersal distances, with longer dispersals occurring with more agricultural land use in potential dispersal paths (an interaction with season was not statistically significant; Fig. 5, Table S7). Dispersal distances also increased as the number of proximate individuals (per available) decreased (Fig. 5, Table S7). Our sensitivity analysis suggests our results are relatively robust to sampling effects, with coefficients estimated from subsampled data falling largely within the confidence intervals for the coefficients estimated from the full data (Figure S6).

Null hypothesis models for log-transformed dispersal distances found evidence for longer dispersal distances with more GPS locations recorded during the focal season (Table S5); however, this is likely explained by the trend toward having more observations in the spring, when dispersals were typically longer, relative to the fall (two-sided t-test: *t* = 1.95, *p* = 0.055). Model results were generally qualitatively robust across all full models (Table S8), with the lone exception being that agricultural land use in potential paths was no longer a statistically significant predictor in the model that included deer body weight. We note, however, that there was large data loss for this model (n = 84 for model without proximity or weight, compared to n = 67 with these covariates included, a reduction of 20.2%; Table S8).

### Dispersal habitat selection

**Fig. 6:**
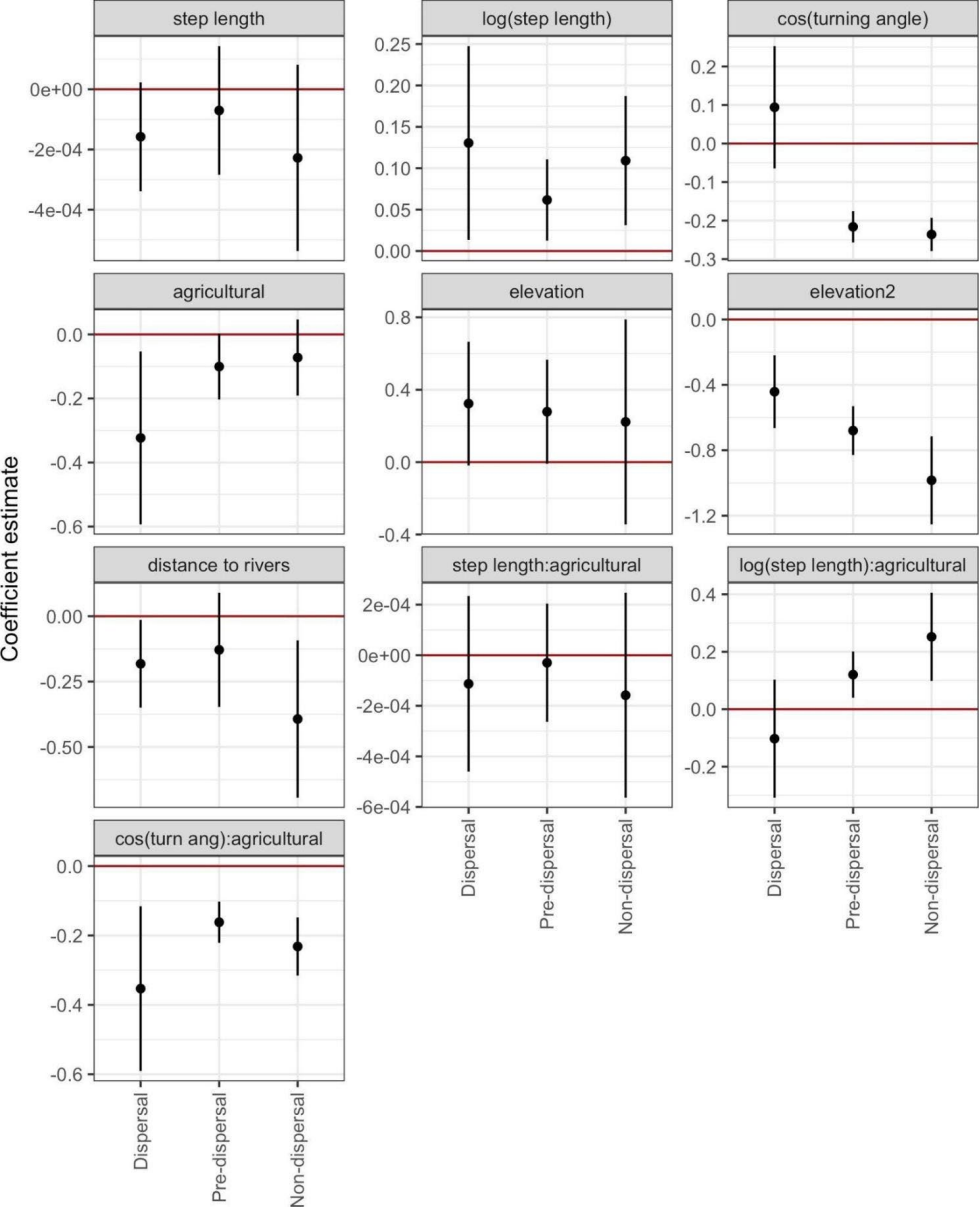
Population-level iSSF coefficient estimates and 95% confidence intervals by movement state (dispersal, pre-dispersal, and non-dispersal movement) for juvenile male white-tailed deer in southwest Wisconsin. Coefficients are not exponentiated such that no selection or avoidance is indicated by a coefficient estimate of 0 (highlighted in red). Note that elevation and elevation2 variables correspond to the second order polynomial for elevation used in models

**Fig. 7:**
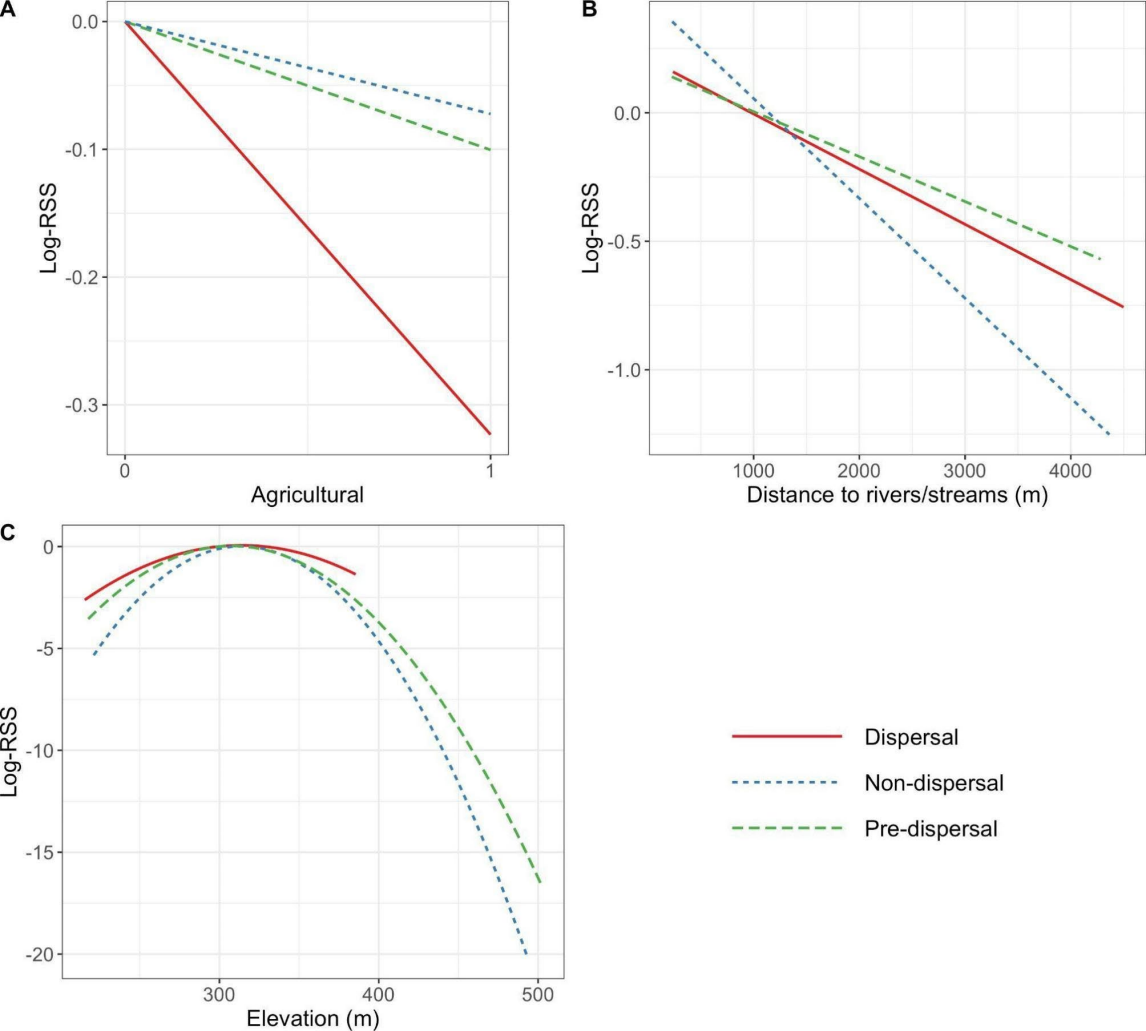
Log-relative selection strength (log-RSS) for the three main habitat covariates in population-level iSSFs for juvenile male white-tailed deer: (A) agricultural land use, (B) distance to nearest river/stream, and (C) elevation. Line types and colors indicate movement state, with dispersal movements in solid red, non-dispersal movements in dotted blue, and pre-dispersal movements in dashed green. Continuous variables are un-scaled and centered, so each line in (B) and (C) has a log-RSS value of zero at the average habitat value for that movement state. Agricultural land use log-RSS is plotted as lines to demonstrate change in selection by movement state. Note that log-RSS was calculated within the bounds of the observed habitat values for a given movement state

Population-level iSSFs showed that dispersing juvenile males avoided agricultural land use and selected for proximity to rivers and streams (Figs. 6 and 7, Table S9). Among the subset of dispersal movements that had adequate road-intersection data (n = 14), juvenile males also avoided intersections with major roads while dispersing (Figure S7, Table S9). Pre-dispersal and non-dispersal movements did not show avoidance of agricultural land use, but non-dispersal movements selected for proximity to rivers and streams (Figs. 6 and 7, Table S9). When repeating iSSFs for dispersal movements with locations recorded every hour, main effects for habitat covariates were largely consistent with models fit to locations recorded every four hours. The key exception was that models fit to hourly locations found dispersing juvenile males selected for intermediate elevations (Figures S8-9, Table S10), which was not found in the four-hour models.

Pre-dispersal and non-dispersal movements tended to have longer step lengths when initiated in agricultural land use, but this was not the case for dispersal movements (Fig. 6, S10, Table S9). In addition, pre- and non-dispersal turning angles were more concentrated (i.e., less variation around moving straight) when steps initiated in agricultural land use. In contrast, when locations were recorded every four hours, dispersal movements were less concentrated in agricultural than non-agricultural land use. However, this relationship disappeared when we evaluated dispersal movements with locations recorded hourly (Figure S11, Tables S9-S10).

Individual-level iSSFs demonstrated inter-individual variability in selection and avoidance patterns, resulting in non-statistically significant habitat selection results when averaged across individuals (Figures S12-S15). However, conclusions regarding the relative selection or avoidance of covariates and differences between movement states were generally robust across population and individual-level iSSF results (Figure S16). In addition, a linear regression for the individual-level agricultural selection coefficient as a function of season and movement state [69] found a significant effect of season (regression coefficient estimate for fall = 0.45, *p* < 0.001). As such, the individual-level results showed a trend across movement states for a seasonal effect of agricultural land use, with weaker avoidance and even potential selection for agricultural land use in the fall (Figure S12), though this pattern was not apparent for dispersal movements recorded at an hourly rate (Figure S17).

## Discussion

We identified dispersal events in 64.2% of juvenile male white-tailed deer, with limited dispersal events in other sex and age classes. Land use—specifically agricultural relative to forested land use—had significant impacts on patterns of dispersal among our juvenile male deer. The probability of dispersal increased with increasing agricultural land use in the natal range, but only for individuals dispersing during the spring. In addition, increased agricultural land use in potential dispersal paths was associated with longer dispersal distances, and individuals tended to avoid agricultural land use during dispersal. In our study area, most of the landscape was classified as either agricultural or forested (for iSSFs, nearly all used and random steps were in one of these two land use classes; used steps: 27.6% in agricultural land use, 69.6% in forested, 1.4% in developed, and 1.4% in other land cover types; Figure S18). As such, our results can reasonably be compared to and are consistent with other studies which focused instead on the relationship between forest cover and dispersal (e.g., lower female dispersal rates with increased forest cover [14] corresponds to our higher male dispersal with increased agricultural land use; see also shorter dispersal distances with increased forest cover [13, 79]).

In the spring, in particular, agricultural land use likely represents low-cover habitat for deer when compared to forested habitat: waste grain is at a minimum and crop heights are low during this period. In the fall, agricultural land use likely serves as effective cover prior to crop harvest and high resource habitat, which, combined with a shift in the social drivers of dispersal (i.e., from intersexual aggression to intrasexual competition for mates [7]), explains the lack of association with dispersal probability in the fall. Indeed, while dispersal movements generally avoided agricultural land use, pre- and non-dispersal movements showed a trend toward reduced avoidance and even selection for agricultural land use during the fall. Dispersal-specific avoidance of agricultural land use in the spring may therefore represent a response to a “landscape of fear” [80] during a high-risk behavior (dispersal) through unfamiliar, low-cover habitat, or even to increased exposure to human activity (e.g., spring planting activities), which can have a stronger impact on cervid behavior than predation [81].

Intersexual aggression from does to offspring is typically implicated as a driver of dispersal in WTD [7]. We lacked data to examine doe-offspring interactions, but competition for forest cover has been suggested as a driver of female dispersal [15], such that agricultural land use may represent lower quality fawning habitat and consequently increase competition for cover in spring. Indeed, such a relationship would explain the positive association we observed between agricultural land use and spring dispersal probability, although we only examined drivers of juvenile male dispersal. The temporally dynamic role of land use in shaping dispersal probability likely reflects the effects of both seasonal variation in landscape features and shifting social drivers of dispersal behavior. Further study of how deer of all age and sex classes use or avoid agricultural land use throughout the year could further refine understanding of the dynamic costs and benefits of these habitats for deer.

Drivers of fall dispersal were poorly explained in our models. Fall dispersal events only occurred in male deer and covered much shorter distances, similar to other studies (e.g., [17]). In discussing shorter fall dispersal distances in WTD, Long et al. [7] argued that mate competition avoidance is likely accomplished over short dispersal distances, while inbreeding avoidance requires longer dispersal distances. As such, our results support the conclusion that fall dispersals reflect short distance shifts to improve access to potential mates [7]. Indeed, regardless of season, we found that dispersal distances were typically shorter with increased numbers of proximate deer. This is in contrast with Diefenbach et al. [79] who argue for modeling deer dispersal distance distributions based on percentage forest cover alone. We do not suggest that our metric for the number of proximate deer is representative of local deer densities, nor that dispersal distances are likely to be shorter with higher deer densities (see instead [15]). Rather, by focusing on an individual’s fine-scale social “neighborhood,” this metric is most representative of the contributions of local social interactions on dispersal distances. Tosa et al. [82] found that juvenile deer appeared to join neighboring social groups when their original social group was experimentally removed, suggesting that deer social environments—and disruption to those environments—can contribute to deer space use and interactions. Further, while we lacked data to examine deer social dynamics by sex, low numbers of male-female interactions are expected to increase fall dispersal rates for juvenile males, while low male-male interactions likely decrease fall dispersal rates [7]. As such, future work may determine if the relationship we observed between the number of proximate deer and dispersal distances is driven by specific inter- or intra-sex interactions to refine understanding of how the social environment of deer contributes to their dispersal and movement ecology [83]. While under- or biased sampling is a concern in studies documenting the number or frequency of animal interactions [60–62, 84], our sensitivity analysis suggests our results are relatively robust to sampling effort. We do, however, still urge caution in their interpretation. Nevertheless, further assessments of how animal social behavior contributes to movement and habitat selection (e.g., [85]) could be particularly important for predicting geographic spread of infectious disease (i.e., CWD) and designing disease surveillance programs (e.g., [86], where juvenile male dispersal is simulated only as a function of forest cover).

Our results also provide insight into the impact of landscape features on dispersal paths. Firstly, we found that longer dispersal distances occurred with higher agricultural land use in potential paths, aligning with results from other studies of deer dispersal in Pennsylvania and Illinois [13, 14]. From a population management perspective, these results support the potential for longer distance gene flow in agricultural areas, though this effect may be non-linear or altered by habitat fragmentation, as large, continuous expanses of agriculture or grasslands impede deer gene flow [87]. We also found that juvenile males selected for proximity to rivers and streams during dispersal, which is consistent with other work [17], suggesting that riparian areas may also help to direct individual dispersal paths. Our finding that dispersal directions were biased largely towards the east and west agree with previous work in our study area with deer population genetics [23, 24, 88]. Further, the Wisconsin River is a major barrier to northward dispersal (we observed only one individual, an adult female, that successfully dispersed across this river), which aligns with work by Blanchong et al. [88]. However, rivers are not always a significant barrier to deer dispersal, as in work by Lang et al. [5] which indicated the Mississippi River was not a barrier to deer gene flow in the upper Midwest.

We observed an apparent avoidance of southward dispersal (Fig. 3) which concurs with previous population genetics research [23]. This observation could be driven, at least in part, by deer avoidance of a major east-west highway (Highway 18) since we observed avoidance of road crossings during juvenile male dispersal. However, pre-dispersal and non-dispersal movements could not be evaluated for avoidance of road crossings because crossings were so rare for these movement states. This implies that juvenile males are most likely to complete major road crossings during dispersal, even though these events are still avoided. Other work with cervids [20–22, 24, 89] and a range of other mammal species (e.g., bobcat, *Lynx rufus* [90]; puma, *Puma concolor* [91]; hedgehogs, *Erinaceus europaeus* L. [92]; pronghorn, *Antilocapra americana* [93]) has implicated major roads as barriers to animal movement, pathogen spread, and host gene flow [94]. Importantly, in our study area, the landscape south of this apparent semipermeable road barrier is heavily agricultural, and dispersal events that crossed this road were relatively long (Fig. 3). Taken together, it is likely that avoidance of both agricultural land use and major road crossings drive the limited southward dispersal of deer we observed here. This finding is important for predicting, for example, geographic bias in infectious disease spread and designing appropriate disease surveillance and management protocols (e.g., assigning disease management zones based on expected pathogen geographic spread potential). Further, these results highlight the importance of considering animal movement barriers in the context of their surrounding habitat quality [95].

The dispersal events we observed here predominantly occurred in juvenile males. When females did disperse in our study, they only did so in the spring and showed a tendency to disperse earlier than males and for shorter distances (compared to males in the spring), though a few females dispersed greater than 30 km. These results were consistent with previous work in Wisconsin [96], but other regions have found much higher rates of dispersal in female deer [14, 15, 18]. We identified dispersal events—perhaps best referred to as range shifts—in older age classes, though these events were rare and typically over short distances. Importantly, rare, long-distance or barrier-crossing movements can be important for population genetics (e.g., [97]) and disease spread [35, 98]. While long-distance movements were uncommon for adults and females in our area, the potential consequences of such movements highlight the importance of interrogating the assumption that dispersal or range shifts occur predominantly in juvenile males [18]. Further, while we focused on successful dispersal events, future work could benefit from identifying and characterizing short-term excursions, which can play an important role in pathogen transmission as well (e.g., [99]).

### Limitations and broader management implications

Our null models suggested that increased GPS locations were associated with increased detection of dispersal events in the spring. As such, spring dispersal rates among juvenile males may be higher than what we detected (i.e., we may have failed to detect some dispersal events), making our estimates conservative. In addition, our step selection analysis was limited by the often short dispersal events we observed, especially because shorter dispersal events were more likely to lack adequate covariate heterogeneity for inclusion in individual or population-level models. Our step selection results therefore are most representative for habitat selection over longer dispersal paths. However, because short dispersal events expose individuals to fewer habitat “choices”—particularly given our study area’s limited habitat types—higher resolution movement data, in the absence of corresponding fine-scale habitat data, may be unlikely to provide novel habitat selection insight for these short distance dispersal events.

While our findings largely align with other studies of WTD dispersal [13, 14, 17, 19, 20, 22], as of yet, there is no comprehensive framework for drivers of deer dispersal across their highly varied habitats, leading to uncertainty in the generalizability of any location-specific study to other regions. This gap in understanding is particularly relevant in the context of continually expanding CWD: there is not yet a clear understanding of the specific role of juvenile dispersal or other long-distance movements in the spread of CWD [87], which could alter the effectiveness of CWD management. For example, if the local social environment contributes to dispersal distances—and dispersal events are high risk for CWD spread—limiting geographic spread of CWD may benefit from selective removal of social groups, rather than general deer density reduction [82, 100]. A broader-scale (e.g., regional) analysis of long-distance deer movements may therefore improve predictions of CWD spread and response to management interventions.

## Conclusion

In this study, we quantified the important role of land use—particularly agricultural land use—in shaping white-tailed deer dispersal rates, distances, and paths. In addition, our results suggest that an individual’s social environment further contributes to deer dispersal ecology. These findings agree with and build on the body of cervid dispersal literature, and provide key information for deer population management and disease control.

## Electronic supplementary material

Below is the link to the electronic supplementary material.

Supplementary Material 1

## Data Availability

The R code used during the current study is available via the Zenodo digital repository (https://doi.org/10.5281/zenodo.7200058). The data that support the findings of this study are available from the Wisconsin Department of Natural Resources but restrictions apply to the availability of these data, which were used under a data sharing agreement for the current study, and so are not publicly available. Data may be requested from the Wisconsin Department of Natural Resources.

## Abbreviations

aKDE: autocorrelated kernel density estimation.
BAM: butorphanol, azaperone, medetomidine.
CWD: chronic wasting disease.
DOP: dilution of precision.
GPS: global positioning system.
HMM: hidden Markov model.
iSSF: integrated step selection function.
KDE: kernel density estimation.
Log-RSS: log-relative selection strength.
NLCD: National Land Cover Database.
SSF: step selection function.
UDOI: utilization distribution overlap index.
WDNR: Wisconsin Department of Natural Resources.
WTD: white-tailed deer.

## References

[1] Gaines MS, McClenaghan LR (1980). Dispersal in small mammals. Annu Rev Ecol Syst.

[2] Slarkin M (1985). Gene flow in natural populations. Annu Rev Ecol Syst.

[3] Murray BG (1967). Dispersal in Vertebrates Ecology.

[4] Daversa DR, Fenton A, Dell AI, Garner TWJ, Manica A. Infections on the move: how transient phases of host movement influence disease spread. Proc Biol Sci 2017;284. 10.1098/rspb.2017.1807 PMC5745403 29263283

[5] Lang KR, Blanchong JA (2012). Population genetic structure of white-tailed deer: Understanding risk of chronic wasting disease spread. J Wildl Manage.

[6] Cullingham CI, Merrill EH, Pybus MJ, Bollinger TK, Wilson GA, Coltman DW (2011). Broad and fine-scale genetic analysis of white-tailed deer populations: estimating the relative risk of chronic wasting disease spread. Evol Appl.

[7] Long ES, Diefenbach DR, Rosenberry CS, Wallingford BD (2008). Multiple proximate and ultimate causes of natal dispersal in white-tailed deer. Behav Ecol.

[8] Wolff JO, Lundy KI, Baccus R (1988). Dispersal, inbreeding avoidance and reproductive success in white-footed mice. Anim Behav.

[9] Pusey A, Wolf M (1996). Inbreeding avoidance in animals. Trends Ecol Evol.

[10] Stephen Dobson F (1982). Competition for mates and predominant juvenile male dispersal in mammals. Anim Behav.

[11] Benz RA, Boyce MS, Thurfjell H, Paton DG, Musiani M, Dormann CF (2016). Dispersal Ecology Informs Design of Large-Scale Wildlife Corridors. PLoS ONE.

[12] Baguette M, Blanchet S, Legrand D, Stevens VM, Turlure C (2013). Individual dispersal, landscape connectivity and ecological networks. Biol Rev Camb Philos Soc.

[13] Long ES, Diefenbach DR, Rosenberry CS, Wallingford BD, Grund MD (2005). Forest Cover Influences Dispersal Distance of White-Tailed Deer. J Mammal.

[14] Nixon CM, Mankin PC, Etter DR, Hansen LP, Brewer PA, Chelsvig JE (2007). White-tailed Deer Dispersal Behavior in an Agricultural Environment. Am Midl Nat AM.

[15] Lutz CL, Diefenbach DR, Rosenberry CS (2015). Population density influences dispersal in female white-tailed deer. J Mammal.

[16] Nelson ME, Mech LD (1992). Dispersal in Female White-Tailed Deer. J Mammal.

[17] Clements GM, Hygnstrom SE, Gilsdorf JM, Baasch DM, Clements MJ, Vercauteren KC (2011). Movements of white-tailed deer in riparian habitat: Implications for infectious diseases. J Wildl Manage.

[18] Anderson N (2015). Survival and dispersal of white-tailed deer in the agricultural landscape of east-central Illinois. Wildl Biol Pract.

[19] Lutz CL, Diefenbach DR, Rosenberry CS (2016). Proximate influences on female dispersal in white-tailed deer. J Wildl Manage.

[20] Long ES, Diefenbach DR, Wallingford BD, Rosenberry CS (2010). Influence of roads, rivers, and mountains on natal dispersal of white-tailed deer. J Wildl Manage.

[21] Passoni G, Coulson T, Ranc N, Corradini A, Hewison AJM, Ciuti S (2021). Roads constrain movement across behavioural processes in a partially migratory ungulate. Mov Ecol.

[22] Peterson BE, Storm DJ, Norton AS, Van Deelen TR (2017). Landscape influence on dispersal of yearling male white-tailed deer. J Wildl Manage.

[23] Robinson SJ, Samuel MD, Rolley RE, Shelton P (2013). Using landscape epidemiological models to understand the distribution of chronic wasting disease in the Midwestern USA. Landsc Ecol.

[24] Robinson SJ, Samuel MD, Lopez DL, Shelton P (2012). The walk is never random: subtle landscape effects shape gene flow in a continuous white-tailed deer population in the Midwestern United States. Mol Ecol.

[25] Bauder JM, Anderson CS, Gibbs HL, Tonkovich MJ, Walter WD (2021). Landscape features fail to explain spatial genetic structure in white-tailed deer across Ohio, USA. J Wildl Manage.

[26] U.S. Geological Survey. Expanding distribution of chronic wasting disease. 2022 [cited 2022 Mar 11]. Available from: https://www.usgs.gov/centers/nwhc/science/expanding-distribution-chronic-wasting-disease.

[27] Jennelle CS, Henaux V, Wasserberg G, Thiagarajan B, Rolley RE, Samuel MD (2014). Transmission of chronic wasting disease in Wisconsin white-tailed deer: implications for disease spread and management. PLoS ONE.

[28] Gilbertson MLJ, Brandell EE, Pinkerton ME, Meaux NM, Hunsaker M, Jarosinski D, et al. Cause of death, pathology, and chronic wasting disease status of white-tailed deer mortalities in Wisconsin. J Wildl Dis. *In press.*. 10.7589/JWD-D-21-00202 36288680

[29] Evans TS, Kirchgessner MS, Eyler B, Ryan CW, Walter WD (2016). Habitat influences distribution of chronic wasting disease in white-tailed deer. Jour Wild Mgmt.

[30] Edmunds DR, Albeke SE, Grogan RG, Lindzey FG, Legg DE, Cook WE (2018). Chronic wasting disease influences activity and behavior in white-tailed deer. J Wildl Manage.

[31] Lidicker WZ (1988). Solving the Enigma of Microtine “Cycles.”. J Mammal.

[32] Maehr DS, Land ED, Shindle DB, Bass OL, Hoctor TS (2002). Florida panther dispersal and conservation. Biol Conserv.

[33] Joly DO, Ribic CA, Langenberg JA, Beheler K, Batha CA, Dhuey BJ (2003). Chronic wasting disease in free-ranging Wisconsin White-tailed Deer. Emerg Infect Dis.

[34] Jennelle CS, Samuel MD, Nolden CA, Berkley EA (2009). Deer carcass decomposition and potential scavenger exposure to chronic wasting disease. J Wildl Manage.

[35] Oyer AM, Mathews NE, Skuldt LH (2007). Long-distance movement of a white-tailed deer away from a chronic wasting disease area. J Wildl Manage.

[36] Grear DA, Samuel MD, Langenberg JA, Keane D (2006). Demographic patterns and harvest vulnerability of chronic wasting disease infected white-tailed deer in Wisconsin. J Wildl Manage.

[37] Clover MR (1956). Single-gate deer trap. Calif Fish and Game J.

[38] Ramsey CW (1968). A Drop-Net Deer Trap. J Wildl Manage.

[39] Anderson RG, Nielsen CK (2002). Modified Stephenson Trap for Capturing Deer. Wildl Soc Bull.

[40] Miller BF, Osborn DA, Lance WR, Howze MB, Warren RJ, Miller KV (2009). Butorphanol-azaperone-medetomidine for immobilization of captive white-tailed deer. J Wildl Dis.

[41] Severinghaus CW (1949). Tooth Development and Wear as Criteria of Age in White-Tailed Deer. J Wildl Manage.

[42] Storm DJ, Samuel MD, Rolley RE, Beissel T, Richards BJ, Van Deelen TR (2014). Estimating ages of white-tailed deer: Age and sex patterns of error using tooth wear-and-replacement and consistency of cementum annuli. Wildl Soc Bull.

[43] Adams DM, Blanchong JA (2020). Precision of cementum annuli method for aging male white-tailed deer. PLoS ONE.

[44] Lewis JS, Rachlow JL, Garton EO, Vierling LA (2007). Effects of habitat on GPS collar performance: using data screening to reduce location error. J Appl Ecol.

[45] Bjørneraas K, Moorter B, Rolandsen CM, Herfindal I (2010). Screening global positioning system location data for errors using animal movement characteristics. J Wildl Manage.

[46] Hartigan JA, Wong MA, Algorithm (1979). AS 136: A K-Means Clustering Algorithm. J R Stat Soc Ser C Appl Stat.

[47] Rousseeuw PJ, Silhouettes (1987). A graphical aid to the interpretation and validation of cluster analysis. J Comput Appl Math.

[48] Maechler M, Rousseeuw P, Struyf A, Hubert M, Hornik K, Studer M, et al. Package “cluster.” Dosegljivo na. 2013; Available from: https://cran.microsoft.com/snapshot/2014-10-10/web/packages/cluster/cluster.pdf.

[49] Fieberg J, Kochanny CO, Lanham (2005). Quantifying home-range overlap: the importance of the utilization distribution. J Wildl Manage.

[50] Calenge C. The package adehabitat for the R software: tool for the analysis of space and habitat use by animals. Ecological Modelling. 2006. p. 1035.

[51] Fleming CH, Fagan WF, Mueller T, Olson KA, Leimgruber P, Calabrese JM (2015). Rigorous home range estimation with movement data: a new autocorrelated kernel density estimator. Ecology.

[52] Fitak RR, Johnsen S (2017). Bringing the analysis of animal orientation data full circle: model-based approaches with maximum likelihood. J Exp Biol.

[53] Fitak R, Johnsen S. Package “CircMLE.” Maximum Likelihood Analysis of Circular Data. 2020; Available from: http://cran.uni-muenster.de/web/packages/CircMLE/CircMLE.pdf.

[54] Schnute JT, Groot K (1992). Statistical analysis of animal orientation data. Anim Behav.

[55] R Core Team. R: A Language and Environment for Statistical Computing. Vienna, Austria: R Foundation for Statistical Computing; 2018. Available from: https://www.R-project.org/.

[56] Dewitz J, U.S. Geological Survey. National Land Cover Database (NLCD) 2019 products (ver. 2.0, June 2021). 2021. Available from: https://www.sciencebase.gov/catalog/item/5f21cef582cef313ed940043.

[57] Silva I, Fleming CH, Noonan MJ, Alston J, Folta C, Fagan WF (2022). Autocorrelation-informed home range estimation: A review and practical guide. Methods Ecol Evol.

[58] Long J, Long MJ. Package “wildlifeDI.” cran.hafro.is; 2021; Available from: http://cran.hafro.is/web/packages/wildlifeDI/wildlifeDI.pdf.

[59] Long JA, Nelson TA, Webb SL, Gee KL (2014). A critical examination of indices of dynamic interaction for wildlife telemetry studies. J Anim Ecol.

[60] Silk MJ, Jackson AL, Croft DP, Colhoun K, Bearhop S (2015). The consequences of unidentifiable individuals for the analysis of an animal social network. Anim Behav.

[61] Davis GH, Crofoot MC, Farine DR (2018). Estimating the robustness and uncertainty of animal social networks using different observational methods. Anim Behav.

[62] Gilbertson MLJ, White LA, Craft ME (2021). Trade-offs with telemetry-derived contact networks for infectious disease studies in wildlife. Methods Ecol Evol.

[63] McClintock BT, Michelot T, momentuHMM (2018). R package for generalized hidden Markov models of animal movement. Methods Ecol Evol.

[64] Calabrese JM, Fleming CH, Gurarie E (2016). Ctmm: An r package for analyzing animal relocation data as a continuous-time stochastic process. Methods Ecol Evol.

[65] Johnson JB, Omland KS (2004). Model selection in ecology and evolution. Trends Ecol Evol.

[66] Burnham KP, Anderson DR (2004). Multimodel inference: understanding AIC and BIC in model selection. Sociol Methods Res.

[67] Gelman A, Hill J. Data Analysis Using Regression and Multilevel/Hierarchical Models. Cambridge University Press; 2006.

[68] Gelman Su, Yajima, Hill. Package “arm.” mirror.linux.duke.edu; 2013; Available from: https://mirror.linux.duke.edu/cran/web/packages/arm/arm.pdf.

[69] Thurfjell H, Ciuti S, Boyce MS (2014). Applications of step-selection functions in ecology and conservation. Mov Ecol.

[70] Forester JD, Im HK, Rathouz PJ (2009). Accounting for animal movement in estimation of resource selection functions: sampling and data analysis. Ecology.

[71] Avgar T, Potts JR, Lewis MA, Boyce MS (2016). Integrated step selection analysis: bridging the gap between resource selection and animal movement. Methods Ecol Evol.

[72] Signer J, Fieberg J, Avgar T (2019). Animal movement tools (amt): R package for managing tracking data and conducting habitat selection analyses. Ecol Evol.

[73] Schielzeth H, Forstmeier W (2009). Conclusions beyond support: overconfident estimates in mixed models. Behav Ecol.

[74] Muff S, Signer J, Fieberg J (2020). Accounting for individual-specific variation in habitat-selection studies: Efficient estimation of mixed-effects models using Bayesian or frequentist computation. J Anim Ecol.

[75] Craiu RV, Duchesne T, Fortin D, Baillargeon S (2011). Conditional Logistic Regression With Longitudinal Follow-up and Individual-Level Random Coefficients: A Stable and Efficient Two-Step Estimation Method. J Comput Graph Stat.

[76] Craiu RV, Duchesne T, Fortin D, Baillargeon S, Duchesne MT. Package “TwoStepCLogit.” 2016; Available from: https://cran.pau.edu.tr/web/packages/TwoStepCLogit/TwoStepCLogit.pdf.

[77] Avgar T, Lele SR, Keim JL, Boyce MS (2017). Relative Selection Strength: Quantifying effect size in habitat- and step-selection inference. Ecol Evol.

[78] Fieberg J, Signer J, Smith B, Avgar T (2021). A “How to” guide for interpreting parameters in habitat-selection analyses. J Anim Ecol.

[79] Diefenbach DR, Long ES, Rosenberry CS, Wallingford BD, Smith DR (2008). Modeling distribution of dispersal distances in male white-tailed deer. J Wildl Manage.

[80] Laundré JW, Hernández L. The landscape of fear: ecological implications of being afraid. The Open Ecology. 2010;3.

[81] Ciuti S, Northrup JM, Muhly TB, Simi S, Musiani M, Pitt JA (2012). Effects of humans on behaviour of wildlife exceed those of natural predators in a landscape of fear. PLoS ONE.

[82] Tosa MI, Schauber EM, Nielsen CK (2017). Localized removal affects white-tailed deer space use and contacts. J Wildl Manage.

[83] Koen EL, Tosa MI, Nielsen CK, Schauber EM (2017). Does landscape connectivity shape local and global social network structure in white-tailed deer?. PLoS ONE.

[84] Smith JA, Moody J. Structural Effects of Network Sampling Coverage I: Nodes Missing at Random1. Soc Networks. 2013;35. 10.1016/j.socnet.2013.09.003 PMC3846431 24311893

[85] Webber QMR, Prokopenko CM, Kingdon KA, Turner JW, Vander Wal E. Effects of the social environment on movement-integrated habitat selection. bioRxiv. 2021. Available from: 10.1101/2021.02.11.430740. 10.1186/s40462-024-00502-9 PMC11378598 39238061

[86] Belsare AV, Gompper ME, Keller B, Sumners J, Hansen L, Millspaugh JJ. An agent-based framework for improving wildlife disease surveillance: A case study of chronic wasting disease in Missouri white-tailed deer. Ecol Modell. 2020;417. 10.1016/j.ecolmodel.2019.108919 PMC7079769 32189826

[87] Kelly AC, Mateus-Pinilla NE, Brown W, Ruiz MO, Douglas MR, Douglas ME (2014). Genetic assessment of environmental features that influence deer dispersal: implications for prion-infected populations. Popul Ecol.

[88] Blanchong JA, Samuel MD, Scribner KT, Weckworth BV, Langenberg JA, Filcek KB (2007). Landscape genetics and the spatial distribution of chronic wasting disease. Biol Lett.

[89] Prokopenko CM, Boyce MS, Avgar T (2017). Characterizing wildlife behavioural responses to roads using integrated step selection analysis. J Appl Ecol.

[90] Lee JS, Ruell EW, Boydston EE, Lyren LM, Alonso RS, Troyer JL (2012). Gene flow and pathogen transmission among bobcats (*Lynx rufus*) in a fragmented urban landscape. Mol Ecol.

[91] Wheeler DC, Waller LA, Biek R (2010). Spatial analysis of feline immunodeficiency virus infection in cougars. Spat Spatiotemporal Epidemiol.

[92] Rondinini C, Doncaster CP (2002). Roads as barriers to movement for hedgehogs. Funct Ecol.

[93] Robb BS, Merkle JA, Sawyer H, Beck JL, Kauffman MJ (2022). Nowhere to run: semi-permeable barriers affect pronghorn space use. J Wildl Manage.

[94] Trombulak SC, Frissell CA (2000). Review of ecological effects of roads on terrestrial and aquatic communities. Conserv Biol.

[95] Nielsen CK, Anderson RG, Grund MD (2003). Landscape Influences on Deer-Vehicle Accident Areas in an Urban Environment. J Wildl Manage.

[96] Skuldt LH, Mathews NE, Oyer AM (2008). White-tailed deer movements in a chronic wasting disease area in south-central Wisconsin. J Wildl Manage.

[97] Gustafson KD, Vickers TW, Boyce WM, Ernest HB (2017). A single migrant enhances the genetic diversity of an inbred puma population. R Soc Open Sci.

[98] Breed AC, Field HE, Smith CS, Edmonston J, Meers J (2010). Bats without borders: long-distance movements and implications for disease risk management. EcoHealth.

[99] O’Brien JM, O’Brien CS, MCcarthy C, Carpenter TE (2014). Incorporating foray behavior into models estimating contact risk between bighorn sheep and areas occupied by domestic sheep. Wildl Soc Bull.

[100] Kelly AC, Mateus-Pinilla NE, Douglas M, Douglas M, Brown W, Ruiz MO (2010). Utilizing disease surveillance to examine gene flow and dispersal in white-tailed deer. J Appl Ecol.

