# Supplementary material for: Agricultural land use shapes dispersal in white-tailed deer (Odocoileus virginianus)
Source: Mov Ecol. 2022 Oct 26;10:43. doi: 10.1186/s40462-022-00342-5 (PMC9608933; doi:10.1186/s40462-022-00342-5)
Supplement: Supplementary file 1 — Supplementary Material 1 [file 40462_2022_342_MOESM1_ESM.docx]

**Supplementary materials for “Agricultural land use shapes dispersal in white-tailed deer (*Odocoileus virginianus*)”**

Marie L. J. Gilbertson^1^*, Alison Ketz^1^, Matthew Hunsaker^1^, Dana Jarosinski^2,3^, Wesley Ellarson^2^, Daniel P. Walsh^4^, Daniel J. Storm^2^, Wendy C. Turner^5^

^1^Wisconsin Cooperative Wildlife Research Unit, Department of Forest and Wildlife Ecology, University of Wisconsin–Madison, 1630 Linden Dr., Madison, WI, 53706, USA

^2^Wisconsin Department of Natural Resources, 1300 West Clairemont Ave., Eau Claire, WI 54701

^3^Warnell School of Forestry and Natural Resources, University of Georgia, 180 E Green St., Athens, GA, 30602, USA

^4^U.S. Geological Survey, Montana Cooperative Wildlife Research Unit, University of Montana, 32 Campus Drive NS 205, Missoula, MT, 59812, USA

^5^U.S. Geological Survey, Wisconsin Cooperative Wildlife Research Unit, Department of Forest and Wildlife Ecology, University of Wisconsin–Madison, 1630 Linden Dr., Madison, Wisconsin, 53706, USA

*Corresponding author: [mgilbertson5@wisc.edu](mailto:mgilbertson5@wisc.edu)

**Any use of trade, product, or firm names is for descriptive purposes only and does not imply endorsement by the U.S. Government.**

# METHODS

### Table S1: Data sources used in the analysis of white-tailed deer dispersal in southwest Wisconsin

| **Data description** | **Data layer name** | **Source** |
| --- | --- | --- |
| White-tailed deer GPS collar data | Not applicable | WI DNR |
| Land use classifications | National Land Cover Database 2019 | Multi-Resolution Land Characteristics Consortium (MRLC) |
| Rivers and streams | 24K Hydro Flowlines (perennial flows) | WI DNR Open Data* |
| Major roads | Major roads | WI DNR Open Data* |
| Elevation | Digital Elevation Model (DEM) - 30 meter | WI DNR Open Data* |

*Note: WI DNR = Wisconsin Department of Natural Resources. WI DNR Open Data can be accessed at* [*https://data-wi-dnr.opendata.arcgis.com/*](https://data-wi-dnr.opendata.arcgis.com/)


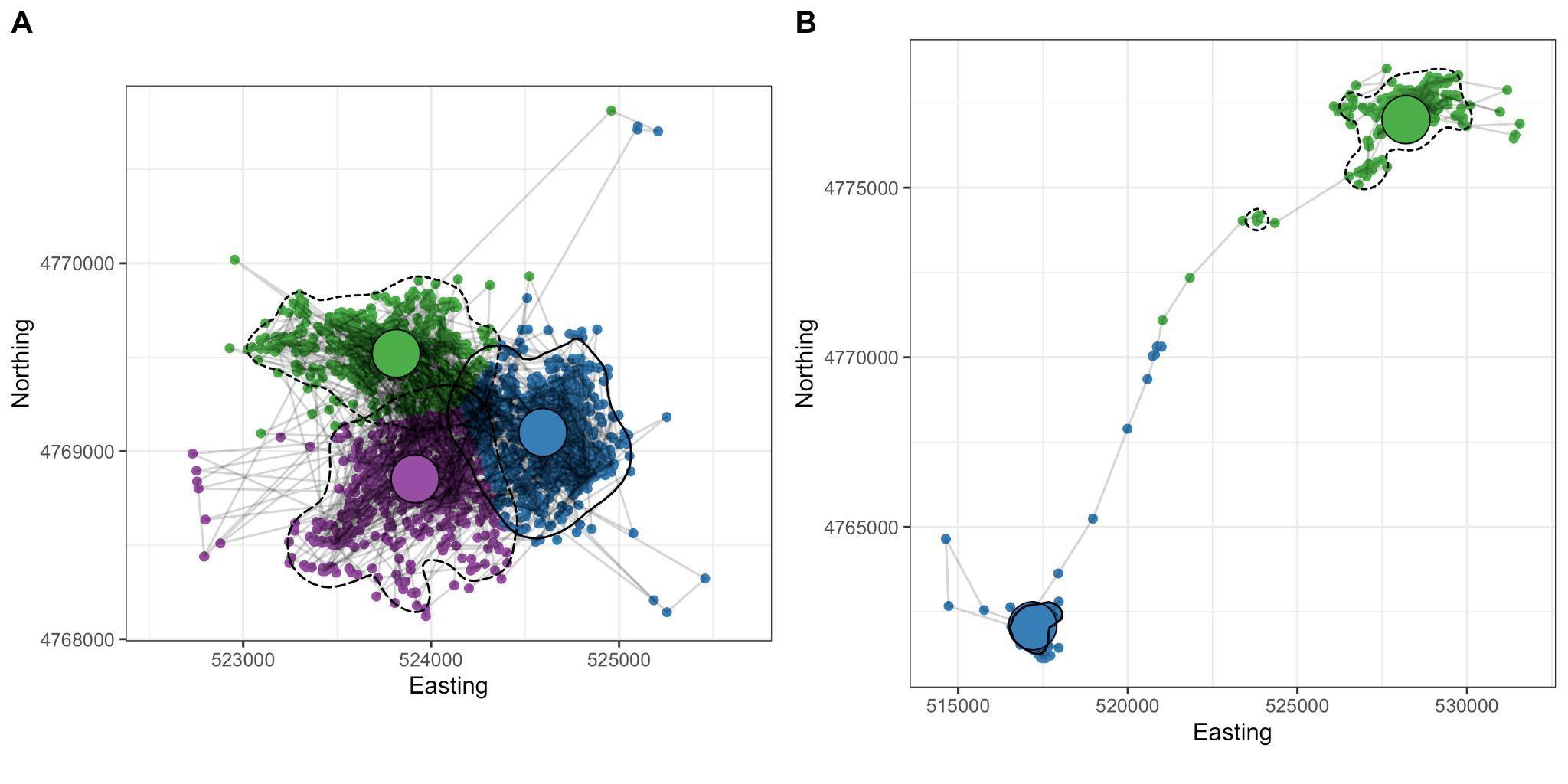


### Figure S1: For dispersal detection in Wisconsin white-tailed deer, example processing of (A) a resident deer, and (B) a dispersing deer (dispersal detection approach described in the main text). Axes are given in meters; small points show individual locations, with sequential locations connected by gray lines. Points are colored according to k-means clustering assignment. Large points indicate k-means cluster centers. Outlines surrounding each k-means cluster show the 95% kernel (KDE) vertices per cluster. In (A) all clusters overlap each other (as determined by utilization distribution overlap index, UDOI), so this individual was classed as a resident. In (B), at least one cluster does not overlap with any other clusters, and the last-used range (green cluster, in this case) is different from and not overlapping with the first used range (blue), so this individual was classed as a disperser.

## Dispersal timing estimation

As stated in the main text, among dispersers, we defined the duration and timing of dispersal as the time from the last GPS location within the pre-dispersal range to the first GPS location within the post-dispersal range. For clusters with at least 30 locations, these ranges were defined as the vertices for the 95% KDE home range from previous *k*-means analysis. If a cluster lacked these vertices (i.e., contained fewer than 30 locations), the range was defined by the 95% minimum convex polygon for that cluster. We chose this definition for dispersal timing based on occasional very short duration dispersals which had few to no recorded GPS locations between ranges. Because GPS locations fell outside the 95% KDE vertices on a number of occasions besides the main dispersal event, we defined a “dispersal window” based on visual inspection of an individual’s net-squared displacement (similar to the approach used by [1]). We then identified the last location in the pre-dispersal range and the first location in the post-dispersal range based on this window. If the range vertices included multiple distinct polygons, we only assessed those that were at least 25% of the size of the largest polygon for that range and contained at least 33% of points for that range (see Figure S2 for examples of dispersal timing estimation). This approach helped reduce underestimating dispersal durations by focusing our timing estimation on core range areas (Figure S2B). As with dispersal classification, all timing estimations were visually inspected for accuracy.


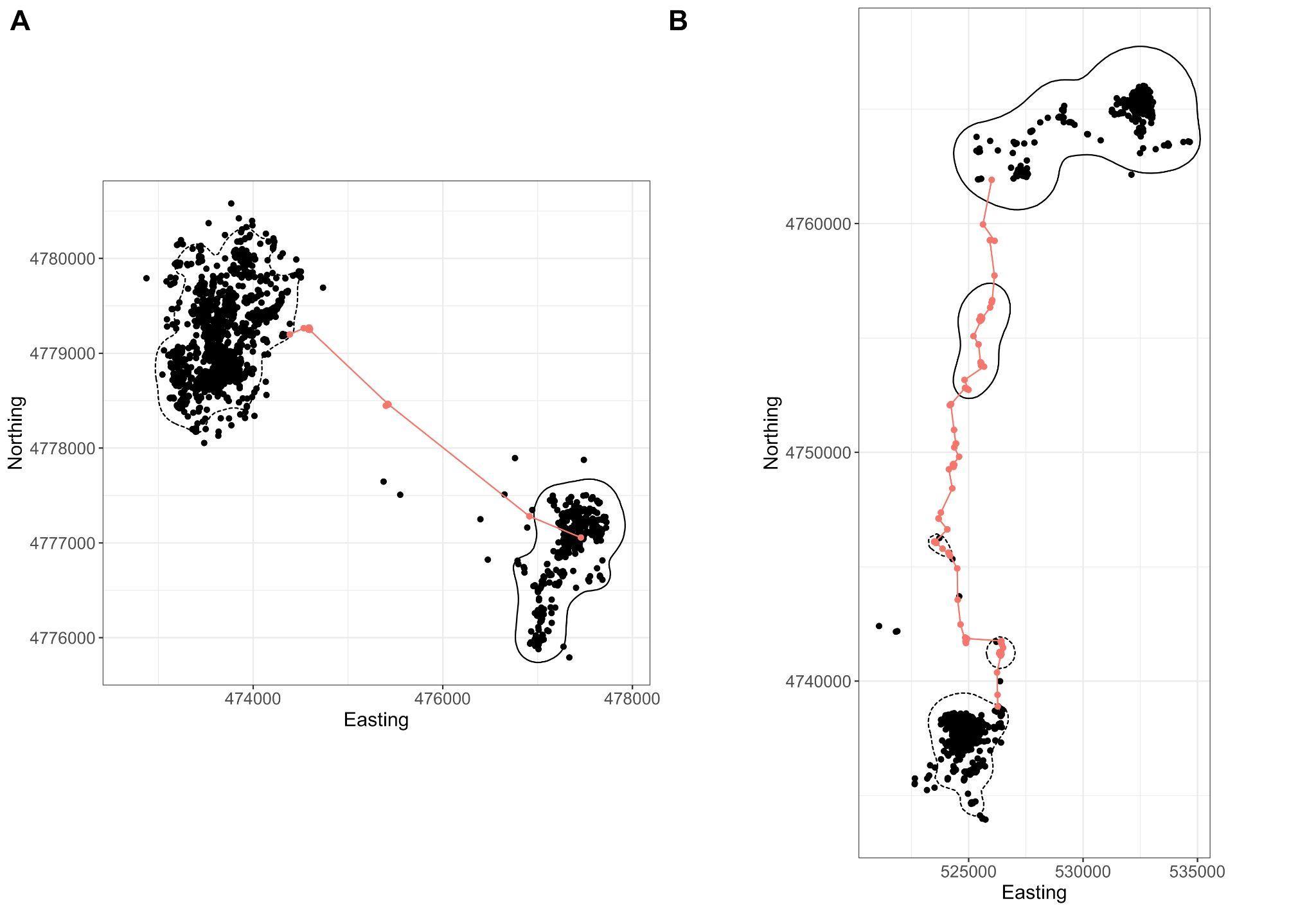


### Figure S2: Examples of estimating timing of dispersal for Wisconsin white-tailed deer, where dispersal paths are shown in pink. Points are GPS locations and the surrounding polygons represent 95% range vertices. Example (A) shows a relatively straightforward but short dispersal event, and example (B) shows an example where multiple polygons were detected as part of the individual’s pre- and post-dispersal ranges, but only large polygons with a high density of points were used for dispersal timing detection (see supplementary methods). Axes give relative easting and northing in meters.

## Autocorrelated kernel density estimation

In order to quantify the proportion of the pre-dispersal range that was classified as agricultural, we performed autocorrelated kernel density estimation (aKDE) to define range boundaries or vertices. As described in the main text, the pre-dispersal range was defined by movements from 1 Mar or 1 Aug to either the date of dispersal (for dispersers) or the median date of dispersal for a given season (for non-dispersers; spring: 25 May; fall: 22 Oct). We then used continuous time movement modeling to generate a “best guess” movement model (see [2]) and estimate 95% home range areas, accounting for autocorrelation (the aKDE; [3]). We simultaneously estimated the standard 95% bivariate normal home range kernel to compare home range area estimates from both KDE and aKDE approaches. With the 95% aKDE home range vertices, we then calculated the proportion of the range that was classified as “agricultural” by National Land Cover Database (NLCD) designations (i.e., pasture or cultivated crops). We compared KDE and aKDE home range sizes with a two-sided paired t-test, finding that range sizes were not significantly different (t = 1.63, *p* = 0.10), and supporting our use of KDE in our dispersal detection workflow for improved efficiency.

## Proximity sensitivity analysis

To determine sensitivity of our *number proximate* metric (number of proximate individuals per available; see main text) to collaring effort, we first fit simple dispersal distance models. These linear regressions modeled log-transformed dispersal distance as a function of season and number proximate with and without an interaction between the two covariates. These models were fit using all available juvenile male disperser data and represent the “best informed model.” To mimic undersampling the deer population, we then randomly subsampled individuals from our movement data to reflect decreasing proportions of the population—90, 70, or 50%—receiving GPS collars. When subsampling, we also allowed for geographic bias in the resulting collar deployments by keeping all individuals within one geographic quadrant (northeast, northwest, southeast, southwest), and sampling individuals from the remaining quadrants to reach the final sample proportion. This subsampling occurred across all individuals (all age and sex classes) to reflect the data used to generate the original number proximate metric. With the resampled data, we then recalculated the number proximate metric (see main text) and re-fit the simple models for dispersal distance. We extracted coefficient estimates and compared them to the estimates from the best informed model. Most coefficient estimates fell within the 95% confidence intervals from the best-informed model (Figure S6), with the vast majority of estimates outside those bounds originating from 50% subsampled populations (especially spatially-biased subsampling). Our inference for the association between number proximate and dispersal distance therefore appears to be fairly robust to subsampling—including geographically biased GPS collar deployment—but we acknowledge that this metric should still be interpreted with caution.

## Integrated step selection analysis

We followed the general guidelines for step selection analyses as described by both Signer *et al* [4] and Fieberg *et al* [5]. Specifically, we kept only those individuals with a median GPS location rate of approximately every four hours (or every one hour for the hourly step selection analysis; see main text). We then resampled movement data for these individuals to include only those steps with a four hour location rate, and kept only locations that were part of at least three consecutive locations (the minimum number needed to calculate a turning angle). We then removed any non-movement steps (i.e., consecutive locations with a step length of zero). Following these processing steps, we kept only those individuals with more than two steps. We used the *amt* package in R [4] to generate random steps for each used step, and extract the habitat covariates associated with each step (see main text). Continuous habitat covariates were scaled and centered for model fitting.

The individuals in the resulting dataset included two that performed two separate dispersal events; for each of these, we included only the longer (greater number of steps) dispersal event. In addition, our non-dispersal movements frequently included movements from the same individuals in both spring and fall. To avoid pseudoreplication in the population-level model, we randomly selected non-dispersing movements such that each individual was represented only once in the dataset.


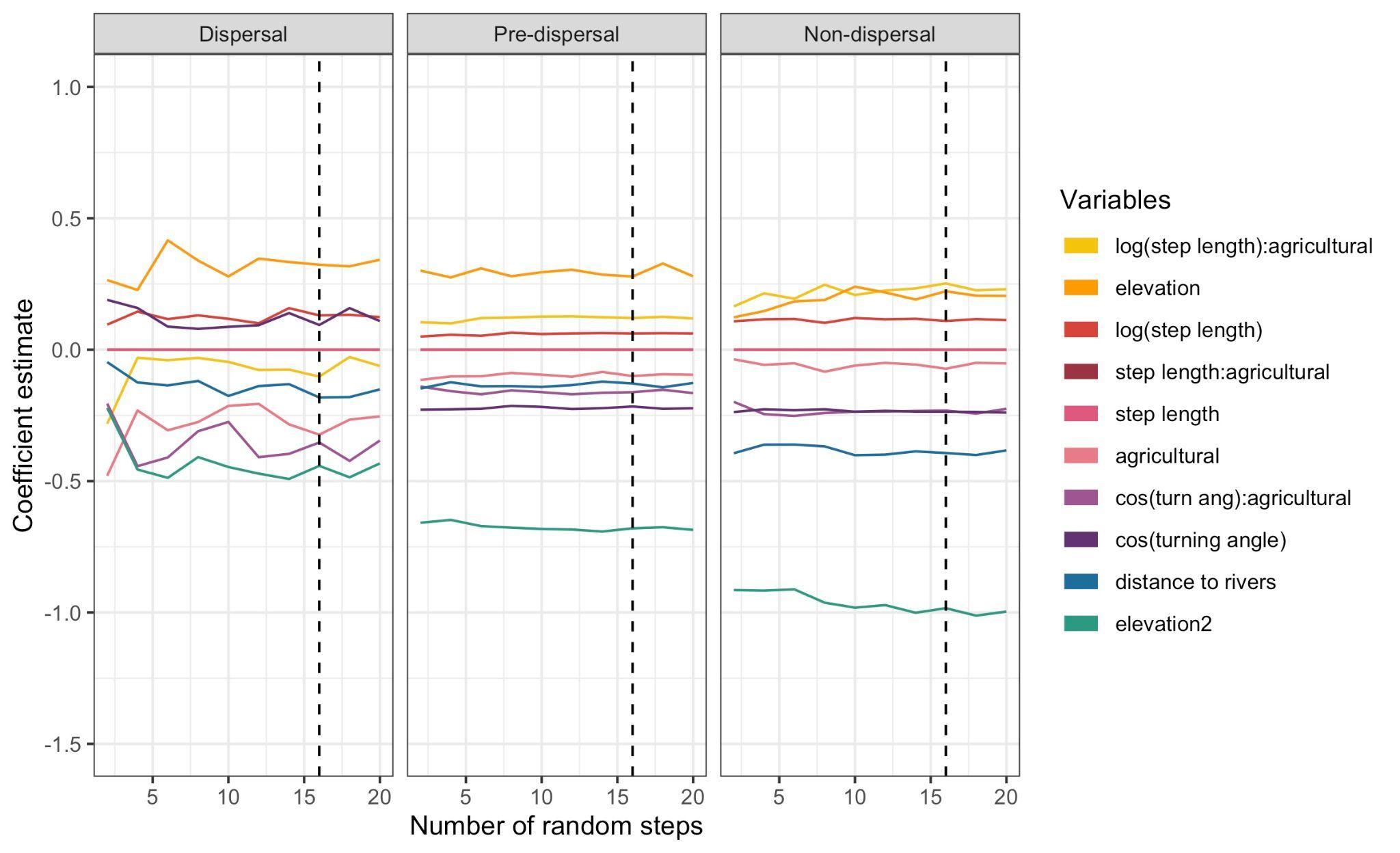


### Figure S3: Integrated step selection function (iSSF) coefficient estimates as the number of random steps increases. Results shown for iSSF models for dispersal, pre-dispersal, and non-dispersal movements. The legend is ordered by coefficient estimates for non-dispersal movements for visibility. The vertical dashed line corresponds to the number of random steps used in the main analyses (16 steps). Note that elevation and elevation2 variables correspond to the second order polynomial for elevation used in models.

# RESULTS

**Table S2: Sample sizes of Wisconsin white-tailed deer by dispersal, sex, and age class**

|  | **Sex** | **Age Class** | | | **Totals** |
| --- | --- | --- | --- | --- | --- |
|  |  | **8mo** | **20mo** | **>2yrs** |  |
| **Dispersed** | **Female** | 6 | 5 | 3 | 14 |
|  | **Male** | 88 | 7 | 2 | 97 |
| **No dispersal detected** | **Female** | 113 | 69 | 158 | 340 |
|  | **Male** | 49 | 49 | 41 | 139 |
| **Totals** | | 256 | 130 | 204 | 590 |


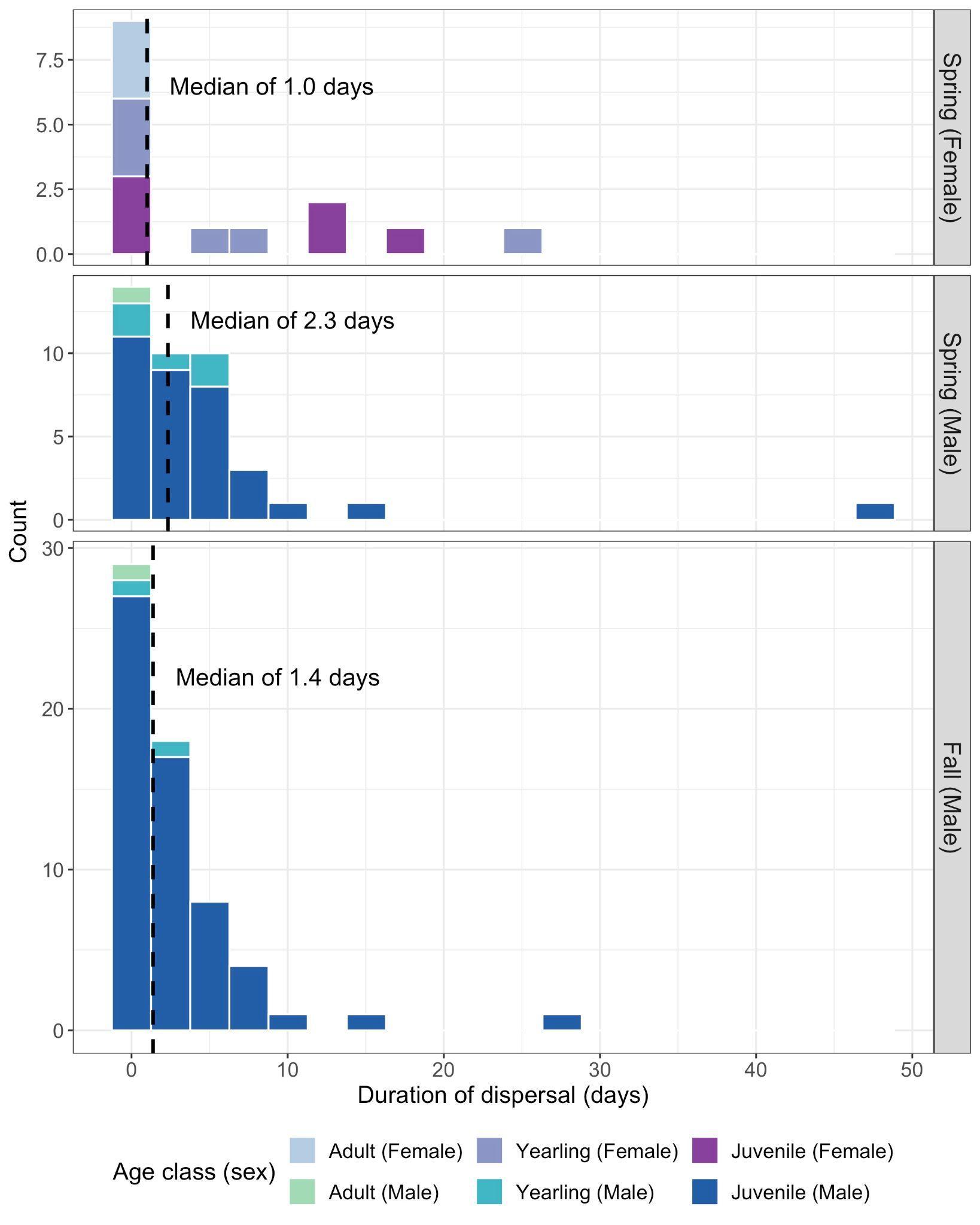


**Figure S4:** Histograms of southwest Wisconsin white-tailed deer dispersal durations (in days), stratified by sex and season. Bars are colored by the age class of individuals at dispersal. Females only dispersed in the spring, and are shown in the top panel; male dispersal durations in the spring and fall are shown in the middle and bottom panels, respectively. Y-axes vary between panels, with the bottom panel the largest due to its higher count values. Median dispersal durations per season and sex are shown with vertical dashed lines. Note that individuals that dispersed two times (n = 6) are shown twice to show the full distribution of dispersal durations.

**
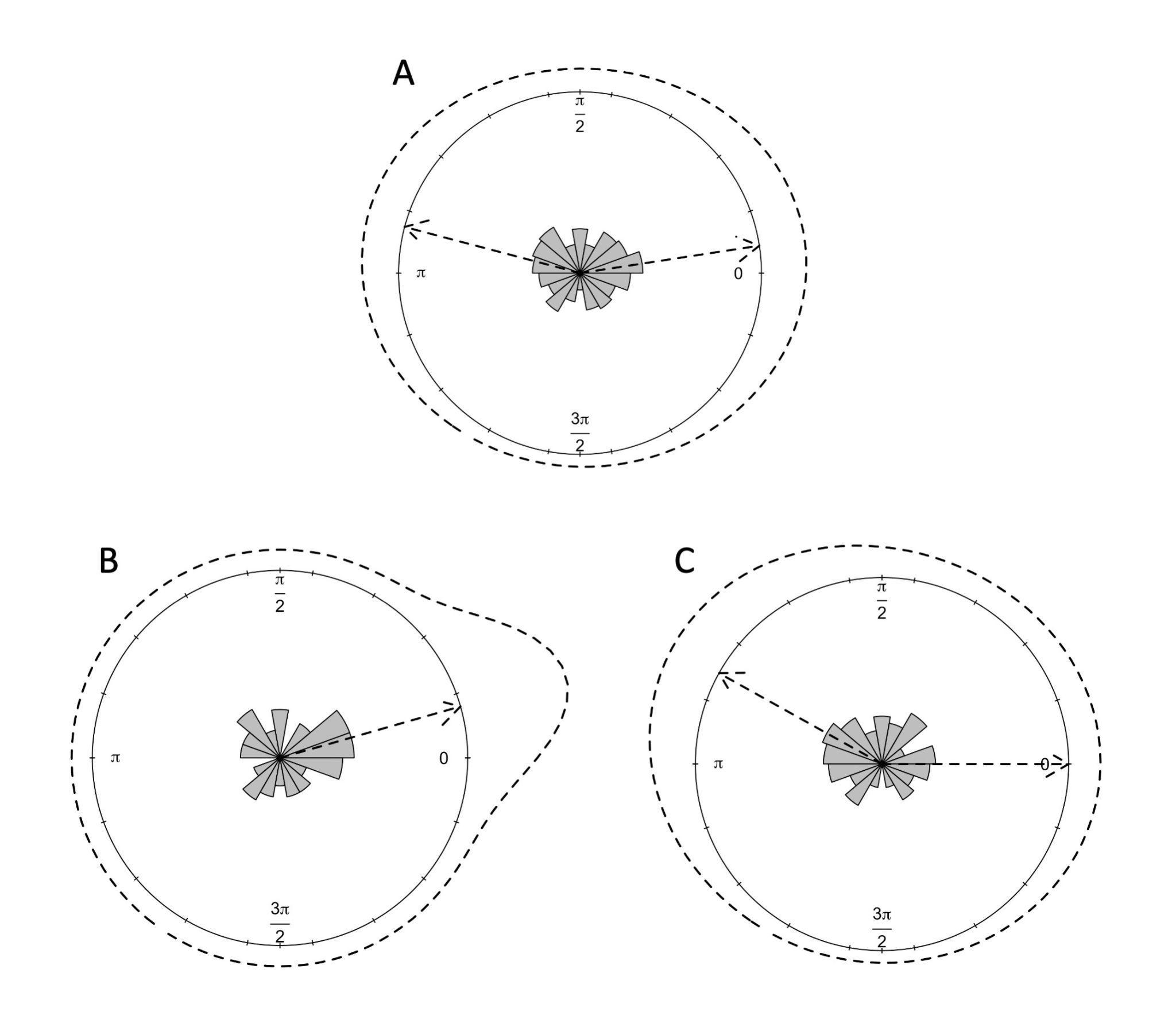
**

### Figure S5: Best models for directionality of southwest Wisconsin white-tailed deer dispersal for (A) all dispersals, (B) spring dispersals only, and (C) fall dispersals only. Central histograms show the distribution of the observed dispersal directions; the density and mean directions of the top model are shown with dashed circles and arrows, respectively. Note that for individuals with multiple dispersals, each dispersal was treated as an independent event here.

###

### Table S3: Circular modeling results for Wisconsin white-tailed deer dispersal direction

| **Dispersal events** | **Model code** | **Model name** | **q1** | **k1** | **lamda** | **q2** | **k2** | **AIC** | **∆AIC** |
| --- | --- | --- | --- | --- | --- | --- | --- | --- | --- |
| All | **M5A** | **Homogenous bimodal** | **0.15** | **1.54** | **0.55** | **2.88** | **1.54** | **423.7** | **0** |
|  | **M5B** | **Bimodal** | **2.67** | **0.48** | **0.75** | **0.17** | **6.08** | **424.3** | **0.60** |
|  | **M3A** | **Homogenous symmetric bimodal** | **6.26** | **1.46** | **0.50** | **9.41** | **1.46** | **424.8** | **1.09** |
|  | **M3B** | **Symmetric bimodal** | **0.05** | **1.82** | **0.50** | **3.19** | **1.15** | **425.6** | **1.84** |
|  | M4B | Axial bimodal | 3.31 | 0.37 | 0.75 | 6.46 | 5.96 | 425.7 | 2.02 |
|  | M4A | Homogenous axial bimodal | 0.01 | 1.45 | 0.55 | 3.15 | 1.45 | 426.1 | 2.34 |
|  | M2C | Modified unimodal | 0.24 | 4.18 | 0.25 | NA | 0 | 428.8 | 5.06 |
|  | M2A | Unimodal | 1.05 | 0.28 | 1 | NA | 0 | 429.6 | 5.82 |
|  | M2B | Symmetric modified unimodal | 0.85 | 0.53 | 0.5 | NA | 0 | 429.9 | 6.18 |
|  | M1 | Uniform | NA | 0 | 1 | NA | 0 | 430.1 | 6.33 |
| Spring | **M2C** | **Modified unimodal** | **0.28** | **15.58** | **0.30** | **NA** | **0** | **154.4** | **0** |
|  | **M4B** | **Axial bimodal** | **3.42** | **0.09** | **0.68** | **6.56** | **14.82** | **156.3** | **1.93** |
|  | M2B | Symmetric modified unimodal | 0.28 | 11.87 | 0.5 | NA | 0 | 156.7 | 2.26 |
|  | M3B | Symmetric bimodal | 3.42 | 0.36 | 0.5 | 6.56 | 10.80 | 157.3 | 2.93 |
|  | M5B | Bimodal | 0.28 | 14.83 | 0.32 | 3.16 | 0.10 | 158.3 | 3.92 |
|  | M2A | Unimodal | 0.31 | 0.58 | 1 | NA | 0 | 162.3 | 7.95 |
|  | M4A | Homogenous axial bimodal | 0.29 | 1.34 | 0.73 | 3.43 | 1.34 | 162.6 | 8.25 |
|  | M5A | Homogenous bimodal | 0.28 | 1.35 | 0.73 | 3.37 | 1.35 | 164.6 | 10.24 |
|  | M1 | Uniform | NA | 0 | 1 | NA | 0 | 165.4 | 11.01 |
|  | M3A | Homogenous symmetric bimodal | 3.40 | 1.49 | 0.5 | 6.55 | 1.49 | 165.4 | 11.05 |
| Fall | **M5A** | **Homogenous bimodal** | **6.28** | **1.47** | **0.36** | **2.63** | **1.47** | **220.0** | **0** |
|  | **M5B** | **Bimodal** | **0.31** | **1.47** | **0.44** | **2.80** | **1.94** | **221.0** | **0.97** |
|  | **M2A** | **Unimodal** | **2.08** | **0.49** | **1** | **NA** | **0** | **221.1** | **1.12** |
|  | **M2B** | **Symmetric modified unimodal** | **2.31** | **1.03** | **0.5** | **NA** | **0** | **221.5** | **1.51** |
|  | M4A | Homogenous axial bimodal | 5.97 | 1.43 | 0.35 | 9.11 | 1.43 | 222.4 | 2.43 |
|  | M3B | Symmetric bimodal | 5.97 | 0.77 | 0.5 | 9.11 | 2.04 | 222.5 | 2.46 |
|  | M2C | Modified unimodal | 2.76 | 5.42 | 0.25 | NA | 0 | 222.9 | 2.92 |
|  | M3A | Homogenous symmetric bimodal | 3.01 | 1.49 | 0.5 | 6.15 | 1.49 | 223.0 | 2.98 |
|  | M1 | Uniform | NA | 0 | 1 | NA | 0 | 224.2 | 4.21 |
|  | M4B | Axial bimodal | 2.83 | 1.52 | 0.63 | 5.97 | 1.28 | 224.4 | 4.43 |

*Note: Model codes and names correspond to those described in Schnute and Groot [6] and Fitak and Johnsen [7], with the uniform model (M1) the “null” model. The dispersal events column indicates which dispersal directions were evaluated (e.g., spring only). The q1 and q2 columns give mean direction parameters (in radians) for the first and second directional distributions, respectively. The k1 and k2 columns give concentration parameters for the first and second directional distributions, respectively. Lambda is the proportional size of the first distribution. Bolded models are those within 2 ∆AIC of the best model (as identified by AIC). NA = not applicable.*

### Table S4: Main model results for southwest Wisconsin juvenile male white-tailed deer dispersal logistic regression

|  | **Model term** | **Exp(estimate)** | **Std. Error** | **95% CI** |
| --- | --- | --- | --- | --- |
| Spring model | **Intercept** | **0.50** | **0.29** | **(0.27-0.86)** |
|  | Weight (lb) | 1.38 | 0.34 | (0.73-2.9) |
|  | **% Agricultural in pre-dispersal range** | **2.01** | **0.34** | **(1.07-4.1)** |
|  | Number proximate | 1.33 | 0.27 | (0.77-2.3) |
|  | Average first poor location | 0.99 | 0.30 | (0.55-1.79) |
|  | Proportion potential paths intersecting roads | 0.87 | 0.30 | (0.47-1.55) |
| Fall model | Intercept | 0.95 | 0.23 | (0.61-1.49) |
|  | Weight (lb) | 0.87 | 0.24 | (0.53-1.39) |
|  | % Agricultural in pre-dispersal range | 1.30 | 0.25 | (0.8-2.16) |
|  | Number proximate | 1.07 | 0.25 | (0.65-1.78) |
|  | Average first poor location | 0.99 | 0.24 | (0.61-1.61) |
|  | Proportion potential paths intersecting roads | 1.16 | 0.25 | (0.71-1.93) |

*Note: All predictors were scaled and centered. Coefficient estimates have been exponentiated. Statistically significant results are highlighted with bold text. Results here correspond with those presented in Figure 4 in the main text. Std. Error = standard error; 95% CI = 95% confidence interval.*

### Table S5: Null model results for southwest Wisconsin juvenile male white-tailed deer dispersal probability and dispersal distance

|  | **model term** | **coefficient estimate** | **std. error** | ***p*-value** |
| --- | --- | --- | --- | --- |
| Spring dispersal probability null models | intercept | -1.03 | 0.37 | **<0.01** |
|  | home range area | 0.65 | 0.32 | **0.041** |
|  | intercept | -0.47 | 0.24 | **0.050** |
|  | number pre-dispersal fixes | 0.58 | 0.26 | **0.023** |
|  | intercept | -0.47 | 0.24 | **0.050** |
|  | longitude of capture | -2.43 | 1.01 | **0.017** |
|  | intercept | -0.18 | 0.43 | 0.67 |
|  | year(2018) | 0.55 | 0.61 | 0.37 |
|  | year(2019) | -0.85 | 0.67 | 0.21 |
|  | year(2020) | -1.28 | 0.77 | 0.10 |
| Fall dispersal probability null models | intercept | 0.37 | 0.32 | 0.25 |
|  | home range area | -0.26 | 0.27 | 0.32 |
|  | intercept | 0.12 | 0.20 | 0.54 |
|  | number pre-dispersal fixes | 0.01 | 0.20 | 0.96 |
|  | intercept | 0.12 | 0.20 | 0.54 |
|  | longitude of capture | -0.73 | 0.93 | 0.43 |
|  | intercept | 0.35 | 0.38 | 0.36 |
|  | year(2018) | -0.23 | 0.62 | 0.71 |
|  | year(2019) | -0.21 | 0.53 | 0.70 |
|  | year(2020) | -0.52 | 0.56 | 0.35 |
| log(dispersal distance) null models | intercept | 1.88 | 0.13 | **<0.001** |
|  | home range area | 0.10 | 0.10 | 0.310 |
|  | intercept | 1.98 | 0.08 | **<0.001** |
|  | number pre-dispersal fixes | 0.24 | 0.08 | **<0.01** |
|  | intercept | 1.98 | 0.09 | **<0.001** |
|  | longitude of capture | -0.20 | 0.36 | 0.590 |
|  | intercept | 1.66 | 0.14 | **<0.001** |
|  | year(2018) | 0.62 | 0.21 | **0.004** |
|  | year(2019) | 0.21 | 0.22 | 0.349 |
|  | year(2020) | 0.59 | 0.24 | **0.017** |
|  | intercept | 2.50 | 0.12 | **<0.001** |
|  | season(fall) | -0.84 | 0.15 | **<0.001** |

*Note: Coefficients are not exponentiated. Statistically significant results are highlighted in bold. Each broad class of model (e.g., spring dispersal probability models) includes several null models, delineated by outlining borders. Std. error = standard error.*

### Table S6: Coefficient estimates across full logistic regressions for southwest Wisconsin juvenile male white-tailed deer dispersal probability

|  | **Term** | **Model 1** | **Model 2a** | **Model 2b** | **Model 3** |
| --- | --- | --- | --- | --- | --- |
| Spring dispersal | Number of individuals in model (n dispersers) | 79 (31) | 74 (28) | 74 (28) | 65 (23) |
|  | Intercept | **0.56^*^** | **0.56^*^** | **0.55^*^** | **0.50^*^** |
|  | Weight (lb) | NA | NA | NA | 1.38 |
|  | Proportion agricultural in pre-dispersal range | **2.03^*^** | **2.54^**^** | **2.50^**^** | **2.01^*^** |
|  | Number proximate per available | NA | 0.93^†^ | 1.27 | 1.33 |
|  | Mean first potential step in poor habitat | 0.93 | 0.91 | 0.86 | 0.99 |
|  | Proportion potential paths intersecting roads | 1.11 | 0.79 | 0.77 | 0.87 |
| Fall dispersal | Number of individuals in model (n dispersers) | 98 (52) | 89 (45) | 89 (45) | 80 (39) |
|  | Intercept | 1.14 | 1.02 | 1.02 | 0.95 |
|  | Weight (lb) | NA | NA | NA | 0.87 |
|  | Proportion agricultural in pre-dispersal range | 1.31 | 1.35 | 1.30 | 1.30 |
|  | Number proximate per available | NA | 0.79^†^ | 1.07 | 1.07 |
|  | Mean first potential step in poor habitat | 1.14 | 1.05 | 1.03 | 0.99 |
|  | Proportion potential paths intersecting roads | 1.20 | 1.10 | 1.09 | 1.16 |

*Note: The first row for spring and fall results gives the number of individuals assessed in each model with the number of those classed as dispersers given in parentheses; the remaining rows give exponentiated coefficient estimates for each model. For both spring and fall, model 3 corresponds to the model presented in the main text and Table S3. Bold text indicates statistically significant results (* = <0.05; ** = <0.01). NA’s (not applicable) indicate a term wasn’t included in a given model. For both spring and fall, model 2a included an alternative term for proximity: ^†^ = the coefficient corresponds to the model term for average proximity.*

### Table S7: Main model results for southwest Wisconsin juvenile male white-tailed deer dispersal distance linear regression

|  | **Model term** | **Exp(estimate)** | **Std. Error** | **95% CI** |
| --- | --- | --- | --- | --- |
| Log(Dispersal distance) model | **Intercept** | **2.53** | **0.12** | **(2.29 - 2.78)** |
|  | Proportion paths intersecting rivers/streams | 0.01 | 0.08 | (-0.15 - 0.18) |
|  | Proportion potential paths intersecting roads | 0.12 | 0.09 | (-0.06 - 0.29) |
|  | **Season(Fall)** | **-0.93** | **0.16** | **(-1.26 - -0.61)** |
|  | **Number proximate** | **-0.25** | **0.11** | **(-0.47 - -0.04)** |
|  | **Avg. proportion of steps falling in agriculture** | **0.26** | **0.12** | **(0.02 - 0.5)** |
|  | Season(Fall):Number proximate | 0.03 | 0.16 | (-0.28 - 0.35) |
|  | Season(Fall):Steps in agriculture | -0.25 | 0.16 | (-0.57 - 0.07) |

*Note: All predictors were scaled and centered. Statistically significant results are highlighted with bold text. Results here correspond with those presented in Figure 5 in the main text. Std. Error = standard error; 95% CI = 95% confidence interval.*

### Table S8: Coefficient estimates across full linear models for southwest Wisconsin juvenile male white-tailed deer dispersal distance

| **Term** | **Model 1a** | **Model 1b** | **Model 2a** | **Model 2b** | **Model 3** |
| --- | --- | --- | --- | --- | --- |
| Number of individuals in model | 84 | 78 | 78 | 67 | 67 |
| Intercept | **2.46^***^** | **2.47^***^** | **2.46^***^** | **2.53^***^** | **2.65^***^** |
| Proportion paths intersecting rivers/streams | -0.05 | -0.08 | -0.01 | 0.01 | -0.01 |
| Proportion potential paths intersecting roads | 0.01 | 0.00 | 0.05 | 0.12 | 0.12 |
| Weight (lb) | NA | NA | NA | NA | -0.05 |
| Season(Fall) | **-0.80^***^** | **-0.80^***^** | **-0.78^***^** | **-0.93^***^** | **-1.01^***^** |
| Number proximate per available | NA | NA | 0.03^†^ | **-0.25^*^** | -0.19**^*^** |
| Avg. proportion of steps falling in agriculture | **0.31^**^** | 0.19^‡^ | **0.28^*^** | **0.26^*^** | 0.15 |
| Season(Fall):Number proximate | NA | NA | 0.08^†^ | 0.03 | 0.05 |
| Season(Fall):Steps in agriculture | -0.28**^*^** | -0.07 | -0.27 | -0.25 | -0.15 |

*Note: The first row gives the number of individuals assessed in each model; the remaining rows give coefficient estimates for each model. Model 2b corresponds to the model presented in the main text and Table S6. Bold text indicates statistically significant results (* = <0.05; ** = <0.01; *** = <0.001). Non-bold text with * = <0.1. NA’s (not applicable) indicate a term wasn’t included in a given model. Models 1b and 2a included alternative terms for agricultural land and proximity, respectively: ^†^ = the coefficient corresponds to the model term for average proximity; ^‡^ = the coefficient corresponds to the model term for proportion agricultural land in pre-dispersal range.*


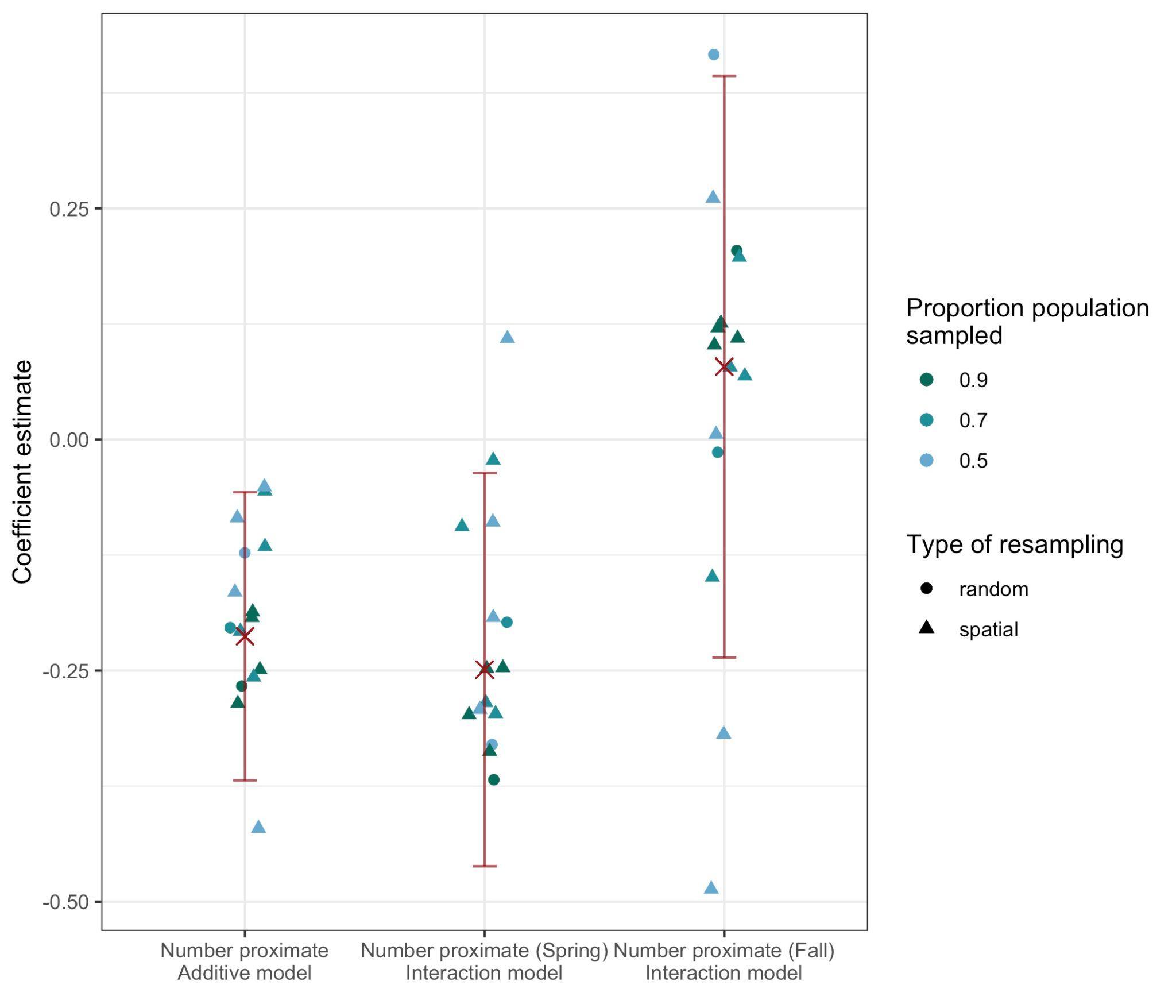


### Figure S6: Coefficient estimates for the number of proximate individuals per available (“Number proximate”) when subsampling Wisconsin white-tailed deer tracking data. Results are shown for modeling the log-transformed juvenile male dispersal distance as a function of the number proximate, with number proximate modeled as an additive variable (“Additive model”) or as an interaction with season (“Interaction model”). Points represent coefficient estimates, with point shape showing the type of resampling (random or spatially biased), and the color the proportion of individuals sampled. The red “x” shows the coefficient estimate with the full data set, with bars showing the 95% confidence intervals with full data.

###

### Table S9: Population-level integrated step selection function (iSSF) estimates by movement state for southwest Wisconsin juvenile male white-tailed deer

|  | **Dispersal** | | **Pre-dispersal** | | **Non-dispersal** | | **Dispersal with roads** | |
| --- | --- | --- | --- | --- | --- | --- | --- | --- |
| **Coefficient** | **Beta Estimate** | **95% CI** | **Beta Estimate** | **95% CI** | **Beta Estimate** | **95% CI** | **Beta Estimate** | **95% CI** |
| step length | -1.58E-04 | (-0.00034 – 0.000023) | -7.04E-05 | (-0.00028 – 0.00014) | -2.28E-04 | (-0.00054 – 0.000082) | -1.50E-05 | (-0.00026 – 0.00023) |
| log(step length) | **1.30E-01** | **(0.014 – 0.25)** | **6.17E-02** | **(0.013 – 0.11)** | **1.09E-01** | **(0.031 – 0.19)** | 7.85E-02 | (-0.097 – 0.25) |
| cos(turning angle) | 9.40E-02 | (-0.065 – 0.25) | **-2.16E-01** | **(-0.26 –**  **-0.18)** | **-2.36E-01** | **(-0.28 –**  **-0.19)** | **2.10E-01** | **(0.029 – 0.39)** |
| agricultural | **-3.23E-01** | **(-0.59 –**  **-0.053)** | -1.00E-01 | (-0.20 – 0.0025) | -7.23E-02 | (-0.19 – 0.047) | -3.66E-01 | (-0.77 – 0.032) |
| elevation | 3.23E-01 | (-0.019 – 0.67) | 2.78E-01 | (-0.0094 – 0.57) | 2.22E-01 | (-0.34 – 0.79) | 2.93E-01 | (-0.15 – 0.73) |
| elevation2 | **-4.42E-01** | **(-0.66 –**  **-0.22)** | **-6.80E-01** | **(-0.83 –**  **-0.53)** | **-9.84E-01** | **(-1.25 –**  **-0.71)** | -1.26E-01 | (-0.37 – 0.12) |
| distance to rivers | **-1.82E-01** | **(-0.35 –**  **-0.014)** | -1.29E-01 | (-0.35 – 0.090) | **-3.93E-01** | **(-0.69 –**  **-0.092)** | -1.90E-01 | (-0.41 – 0.028) |
| step length:agricultural | -1.13E-04 | (-0.00046 – 0.00023) | -2.97E-05 | (-0.00026 – 0.00020) | -1.58E-04 | (-0.00056 – 0.00025) | -5.80E-05 | (-0.00051 – 0.00039) |
| log(step length):agricultural | -1.03E-01 | (-0.31 – 0.10) | **1.20E-01** | **(0.040 – 0.20)** | **2.52E-01** | **(0.099 – 0.41)** | -1.41E-01 | (-0.40 – 0.12) |
| cos(turn ang):agricultural | **-3.53E-01** | **(-0.59 –**  **-0.12)** | **-1.62E-01** | **(-0.22 –**  **-0.10)** | **-2.32E-01** | **(-0.32 –**  **-0.15)** | **-4.66E-01** | **(-0.78 –**  **-0.15)** |
| road intersection | NA | NA | NA | NA | NA | NA | **-6.52E-01** | **(-1.22 –**  **-0.087)** |
| sample size | 33 | | 75 | | 45 | | 14 | |

*Note: Coefficient estimates are un-exponentiated. Statistically significant covariates are highlighted with bold text. The “Dispersal with roads” column gives results for iSSF examining the subset of dispersers with road crossings; all other models did not include the road intersection covariate. Sample sizes per model are given in the bottom row. Elevation and elevation2 variables correspond to the second order polynomial for elevation used in models. 95% CI = 95% confidence interval; NA = not applicable.*


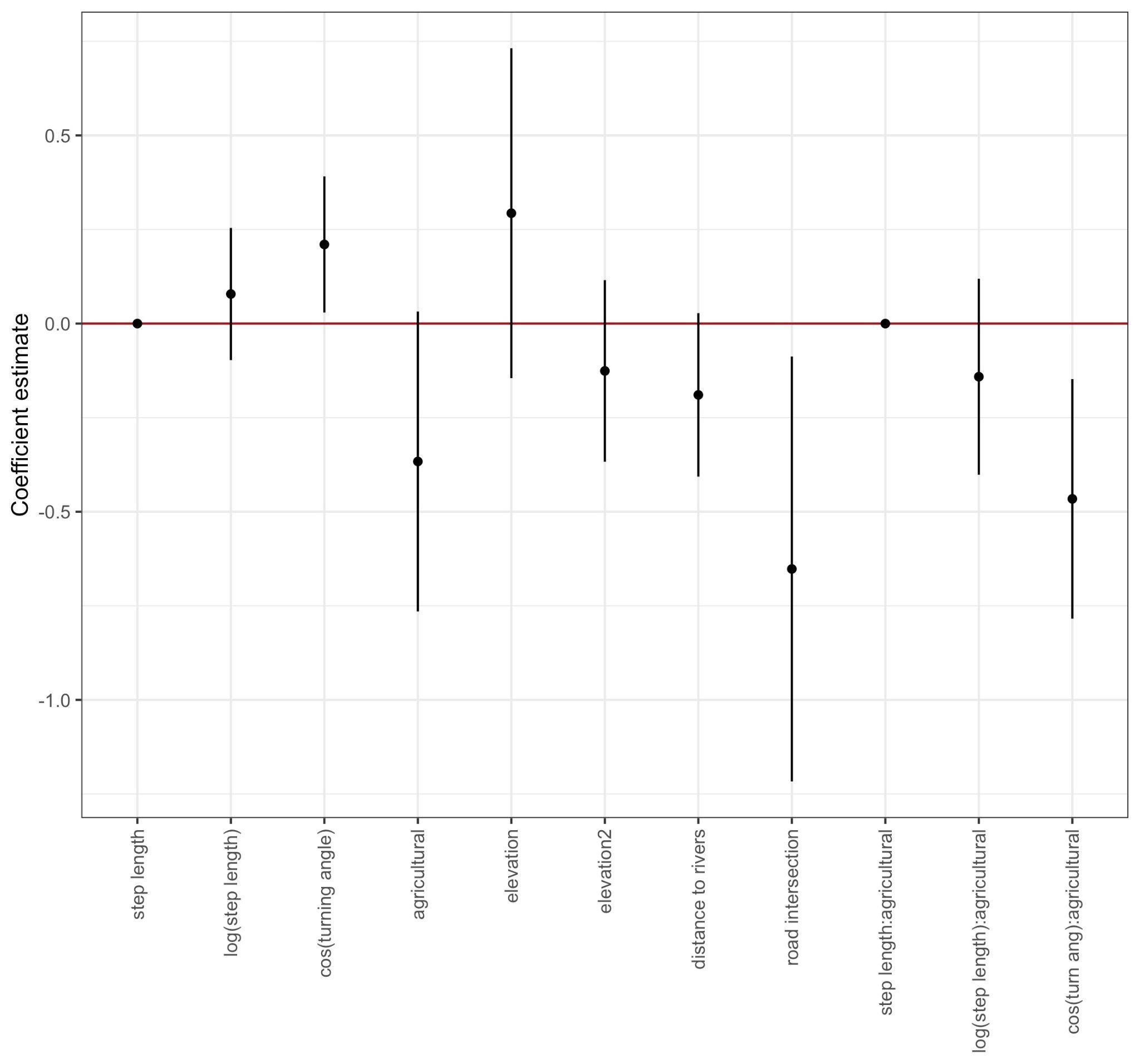


### Figure S7: Population-level iSSF coefficient estimates and 95% confidence intervals for the subset of Wisconsin juvenile male white-tailed deer dispersal movements that included adequate road intersection data (n = 14), and with locations recorded every four hours. Coefficients are not exponentiated such that no selection or avoidance is indicated by a coefficient estimate of 0 (highlighted in red). Note that elevation and elevation2 variables correspond to the second order polynomial for elevation used in models.


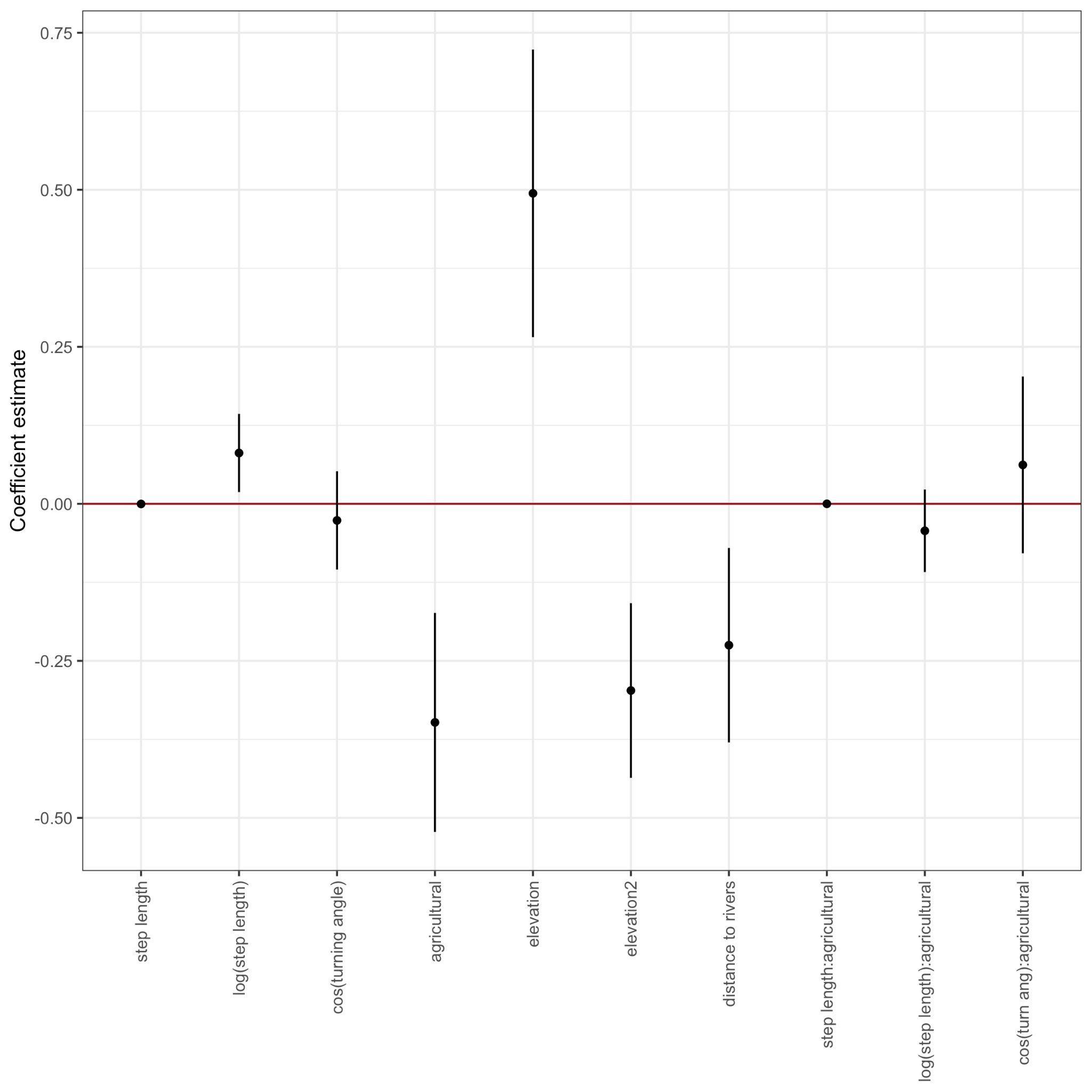


### Figure S8: Population-level iSSF coefficient estimates and 95% confidence intervals for Wisconsin juvenile male white-tailed deer dispersal movements with locations recorded every one hour. Coefficients are not exponentiated such that no selection or avoidance is indicated by a coefficient estimate of 0 (highlighted in red). Note that elevation and elevation2 variables correspond to the second order polynomial for elevation used in models.


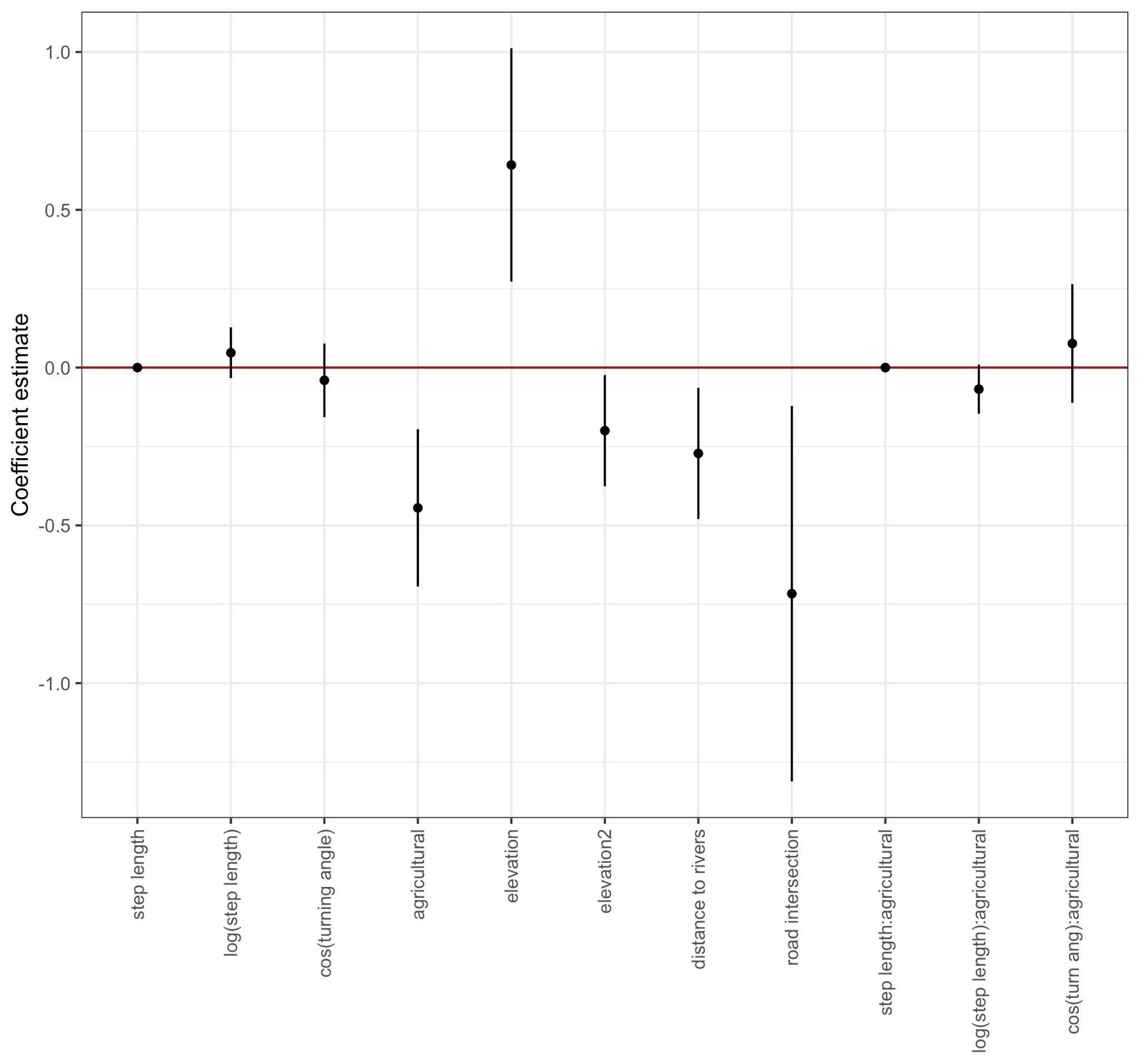


### Figure S9: Population-level iSSF coefficient estimates and 95% confidence intervals for the subset of Wisconsin juvenile male white-tailed deer dispersal movements that included adequate road intersection data (n = 18), and with locations recorded every one hour. Coefficients are not exponentiated such that no selection or avoidance is indicated by a coefficient estimate of 0 (highlighted in red). Note that elevation and elevation2 variables correspond to the second order polynomial for elevation used in models.

### Table S10: Population-level integrated step selection function (iSSF) estimates for southwest Wisconsin juvenile male white-tailed deer dispersal movements with locations recorded every one hour

|  | **Dispersal** | | **Dispersal with roads** | |
| --- | --- | --- | --- | --- |
| **Coefficient** | **Beta Estimate** | **95% CI** | **Beta Estimate** | **95% CI** |
| step length | -1.89E-04 | (-4.2E-04 – 4.02E-05) | 3.48E-06 | (-0.00027 – 0.00028) |
| log(step length) | **8.10E-02** | **(0.019 – 0.14)** | 4.69E-02 | (-0.033 – 0.13) |
| cos(turning angle) | -2.64E-02 | (-0.10 – 0.052) | -4.03E-02 | (-0.16 – 0.076) |
| agricultural | **-3.48E-01** | **(-0.52 – -0.17)** | **-4.45E-01** | **(-0.69 – -0.20)** |
| elevation | **4.94E-01** | **(0.27 – 0.72)** | **6.42E-01** | **(0.27 – 1.012)** |
| elevation2 | **-2.97E-01** | **(-0.44 – -0.16)** | **-2.00E-01** | **(-0.38 – -0.024)** |
| distance to rivers | **-2.25E-01** | **(-0.38 – -0.070)** | **-2.72E-01** | **(-0.48 – -0.064)** |
| step length:agricultural | 4.27E-05 | (-0.00031 – 0.00040) | 1.07E-05 | (-0.00037 – 0.00040) |
| log(step length):agricultural | -4.29E-02 | (-0.11 – 0.023) | -6.82E-02 | (-0.15 – 0.0098) |
| cos(turn ang):agricultural | 6.20E-02 | (-0.079 – 0.20) | 7.63E-02 | (-0.11 – 0.26) |
| road intersection | NA | NA | **-7.17E-01** | **(-1.31 – -0.12)** |
| sample size | 38 | | 18 | |

*Note: Coefficient estimates are un-exponentiated. Statistically significant covariates are highlighted with bold text. The “Dispersal with roads” columns give results for iSSF examining the subset of dispersers with road crossings; other models did not include the road intersection covariate. Sample sizes per model are given in the bottom row. Elevation and elevation2 variables correspond to the second order polynomial for elevation used in models. 95% CI = 95% confidence interval; NA = not applicable.*

**
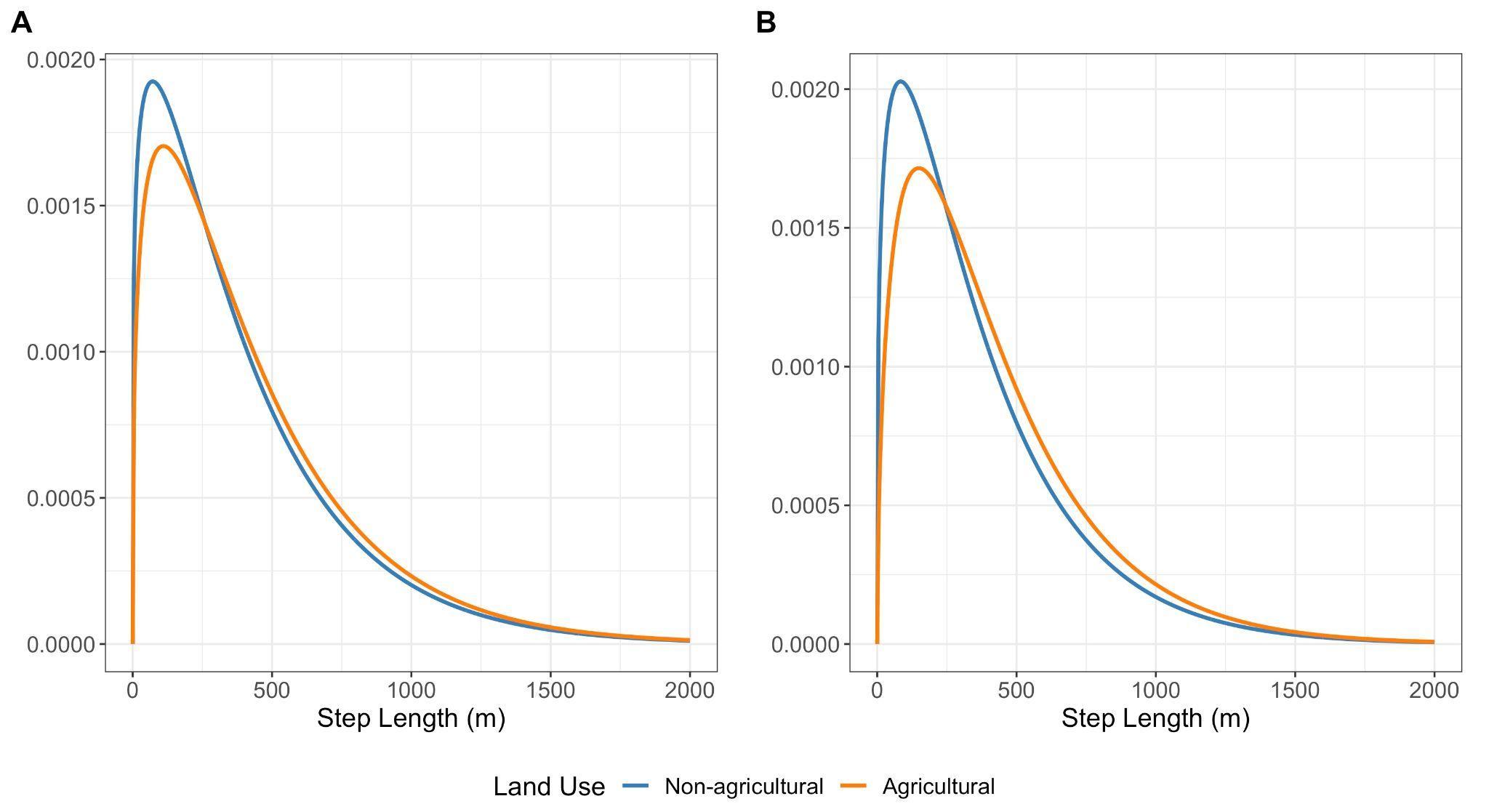
**

### Figure S10: For juvenile male white-tailed deer in southwest Wisconsin, model-predicted step length distributions for (A) pre-dispersal, (B) non-dispersal movements when steps initiated in non-agricultural (blue) versus in agricultural land (orange).


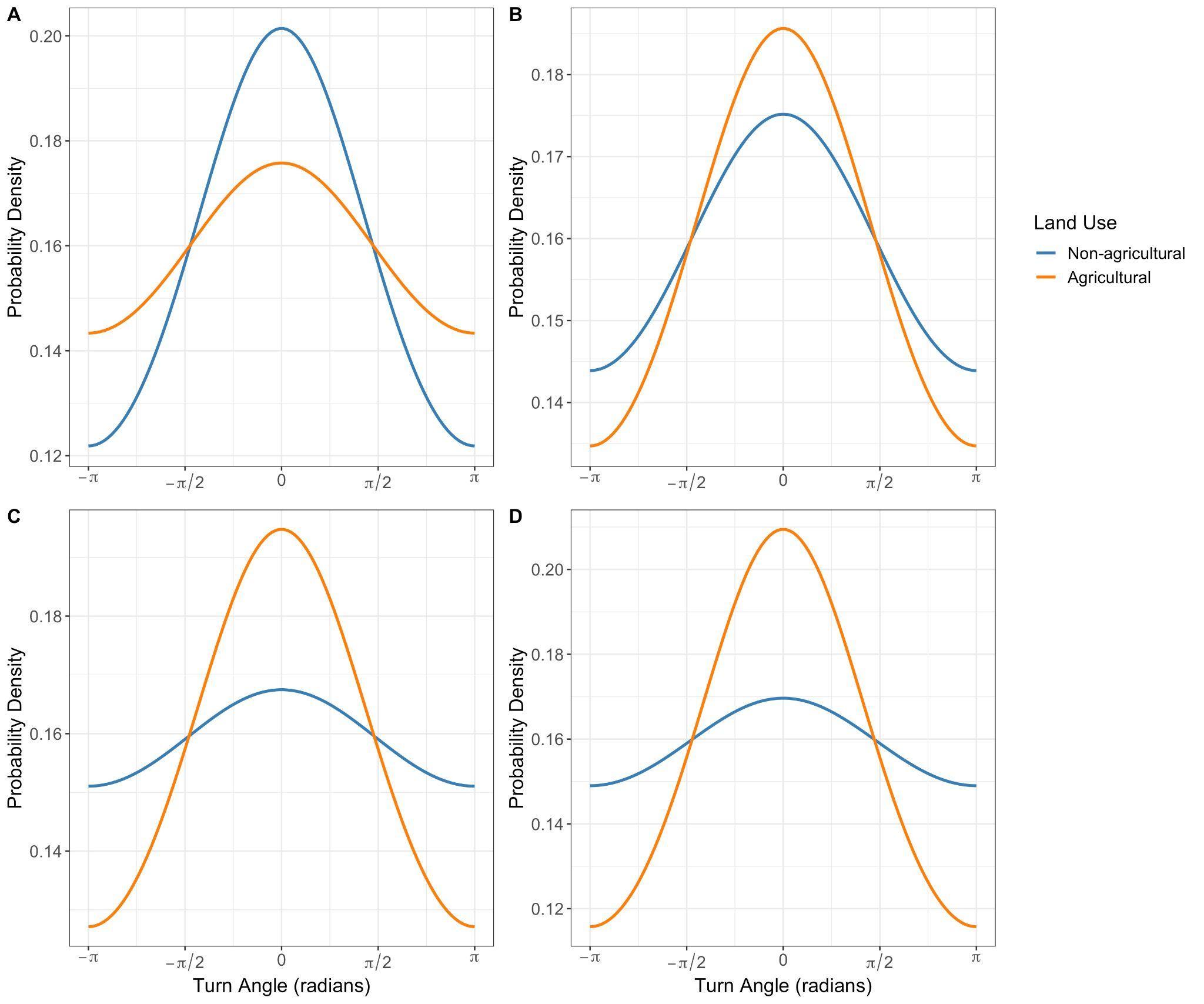


### Figure S11: For juvenile male white-tailed deer in southwest Wisconsin, model predicted turning angle distributions for (A) dispersal movements with locations every four hours, (B) dispersal movements with locations every one hour, (C) pre-dispersal movements, and (D) non-dispersal movements. Distributions are colored by land-use classification at a step’s initiation point, with non-agricultural given in blue and agricultural land in orange.


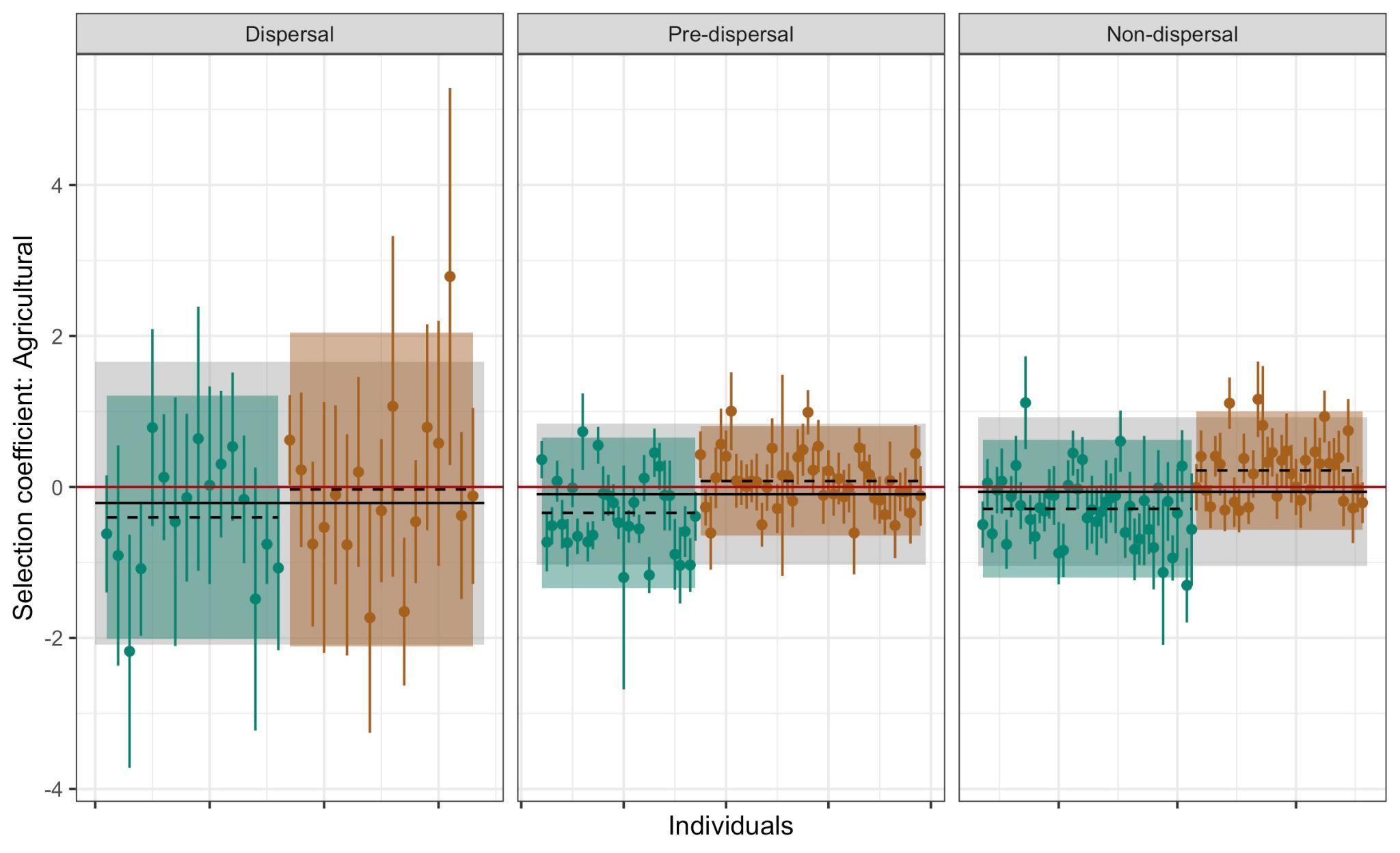


### Figure S12: For juvenile male white-tailed deer in southwest Wisconsin, main effect of agricultural land (un-exponentiated coefficient estimates) from individual-level step selection functions for dispersal (left plot), pre-dispersal (middle plot), and non-dispersal movements (right plot). Points with corresponding vertical lines show individual coefficient estimates and 95% confidence intervals. Colors correspond to seasons, with spring results given in green (left) and fall results in brown (right). The solid black line and background gray box show the mean coefficient estimate and 95% confidence interval (respectively) for each movement state. Dashed lines and colored boxes show the mean coefficient estimate and 95% confidence interval (respectively) stratified by season. The red line highlights a selection coefficient of 0, which corresponds to no selection or avoidance.

###
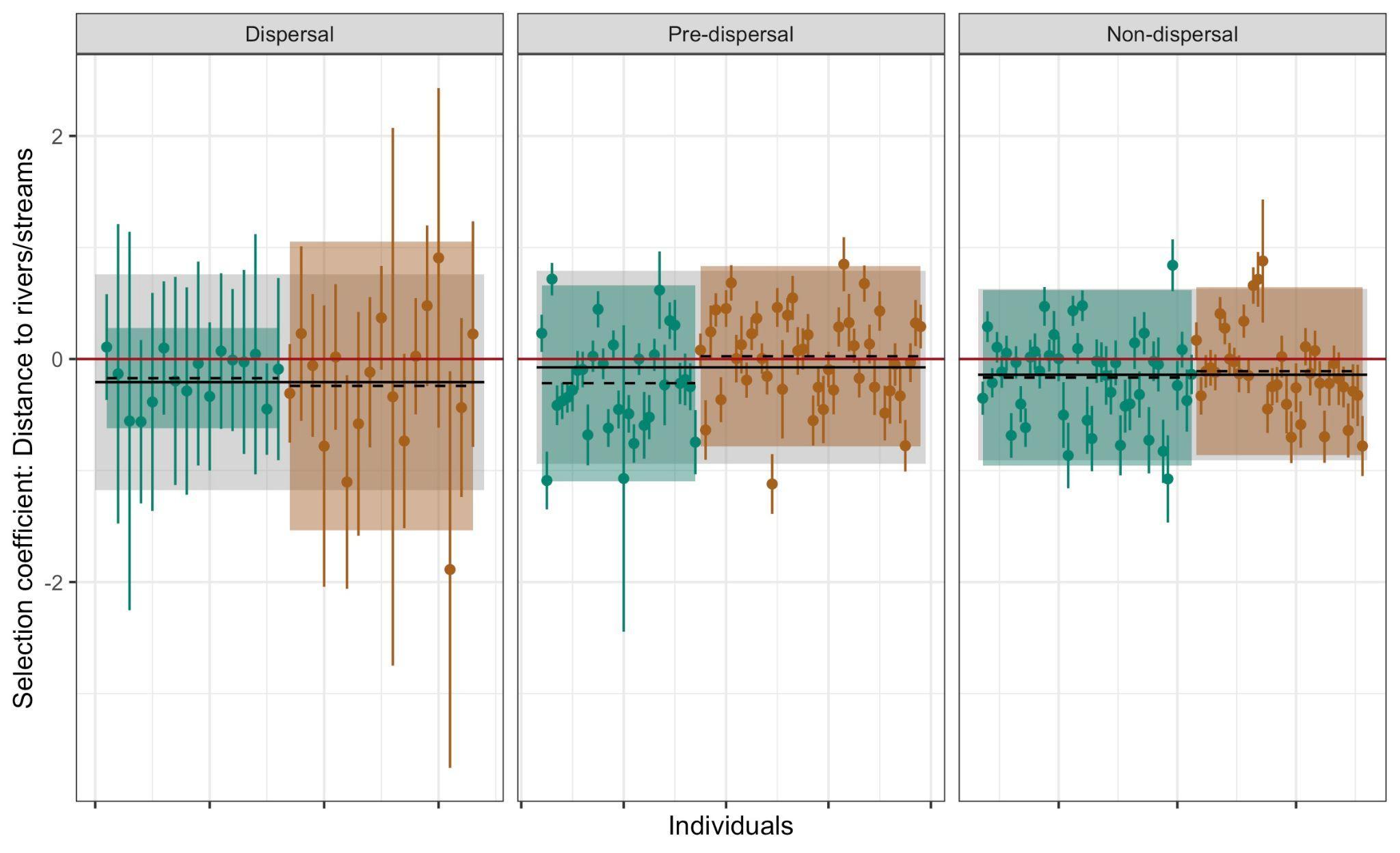
Figure S13: For juvenile male white-tailed deer in southwest Wisconsin, main effect of distance to rivers/streams (un-exponentiated coefficient estimates) from individual-level step selection functions for dispersal (left plot), pre-dispersal (middle plot), and non-dispersal movements (right plot). Points with corresponding vertical lines show individual coefficient estimates and 95% confidence intervals. Colors correspond to seasons, with spring results given in green (left) and fall results in brown (right). The solid black line and background gray box show the mean coefficient estimate and 95% confidence interval (respectively) for each movement state. Dashed lines and colored boxes show the mean coefficient estimate and 95% confidence interval (respectively) stratified by season. The red line highlights a selection coefficient of 0, which corresponds to no selection or avoidance.

###
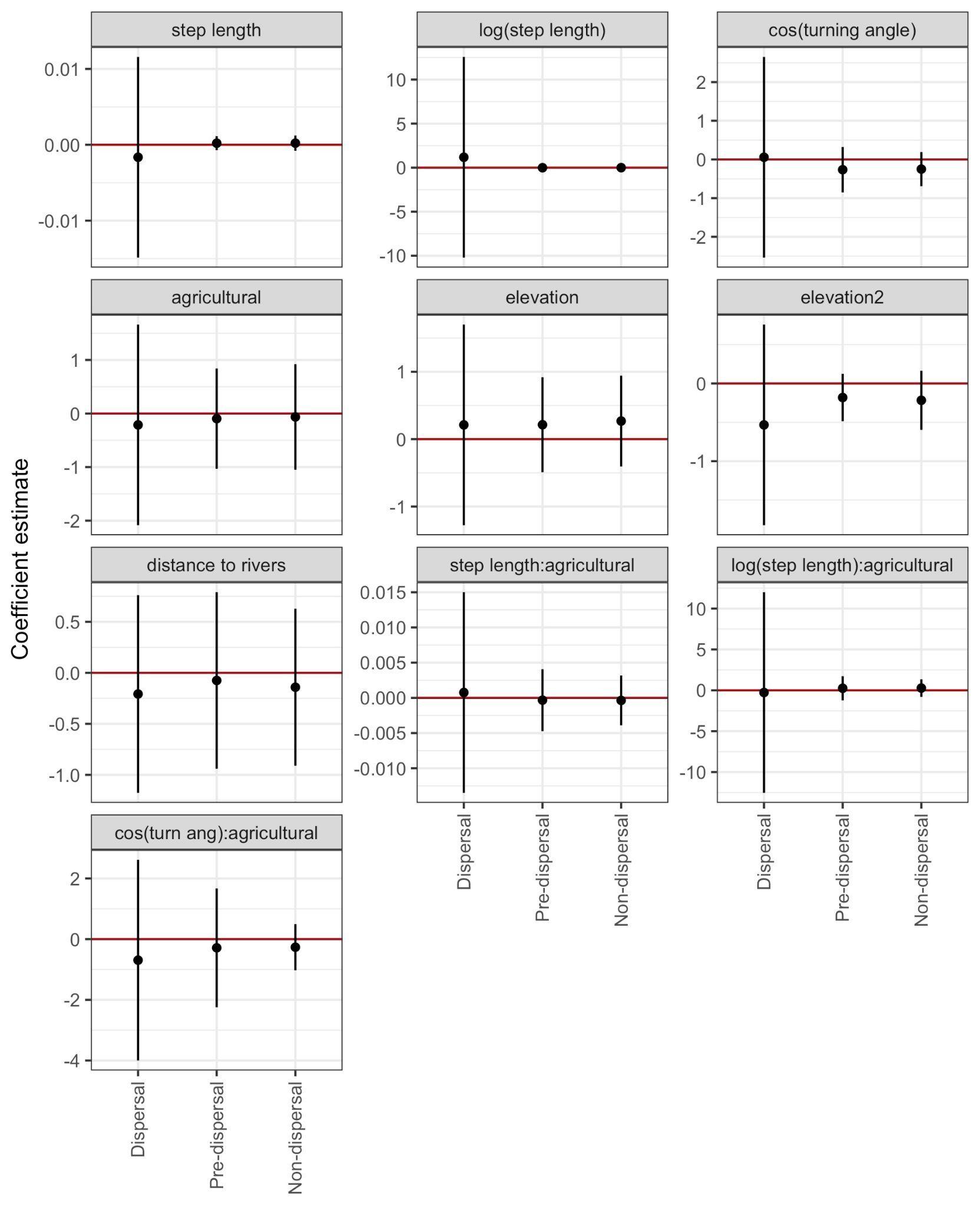
Figure S14: For juvenile male white-tailed deer in southwest Wisconsin, individual-level integrated step selection function (iSSF) mean coefficient estimates and 95% confidence intervals by movement state. Coefficients are not exponentiated such that no selection or avoidance is indicated by a coefficient estimate of 0 (highlighted in red). Note that elevation and elevation2 variables correspond to the second order polynomial for elevation used in models. Intersections with roads results are not shown as models with this variable could only be fit for a small number of individuals and coefficient estimates were therefore highly uncertain.

###
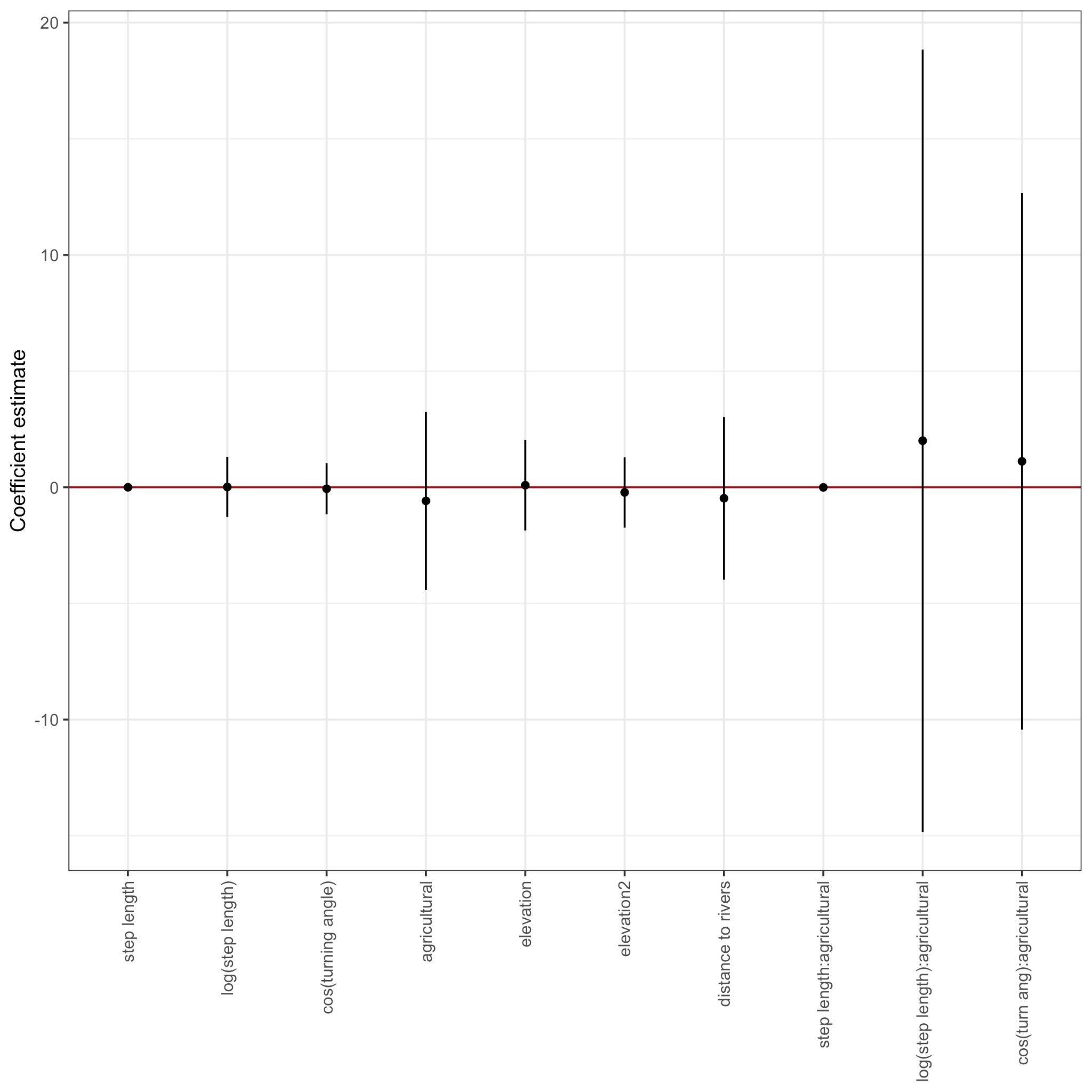
Figure S15: For juvenile male white-tailed deer in southwest Wisconsin, individual-level iSSF mean coefficient estimates and 95% confidence intervals for dispersal movements with locations recorded every one hour. Coefficients are not exponentiated such that no selection or avoidance is indicated by a coefficient estimate of 0 (highlighted in red). Note that elevation and elevation2 variables correspond to the second order polynomial for elevation used in models. Intersections with roads results are not shown as models with this variable could only be fit for a small number of individuals and coefficient estimates were therefore highly uncertain.


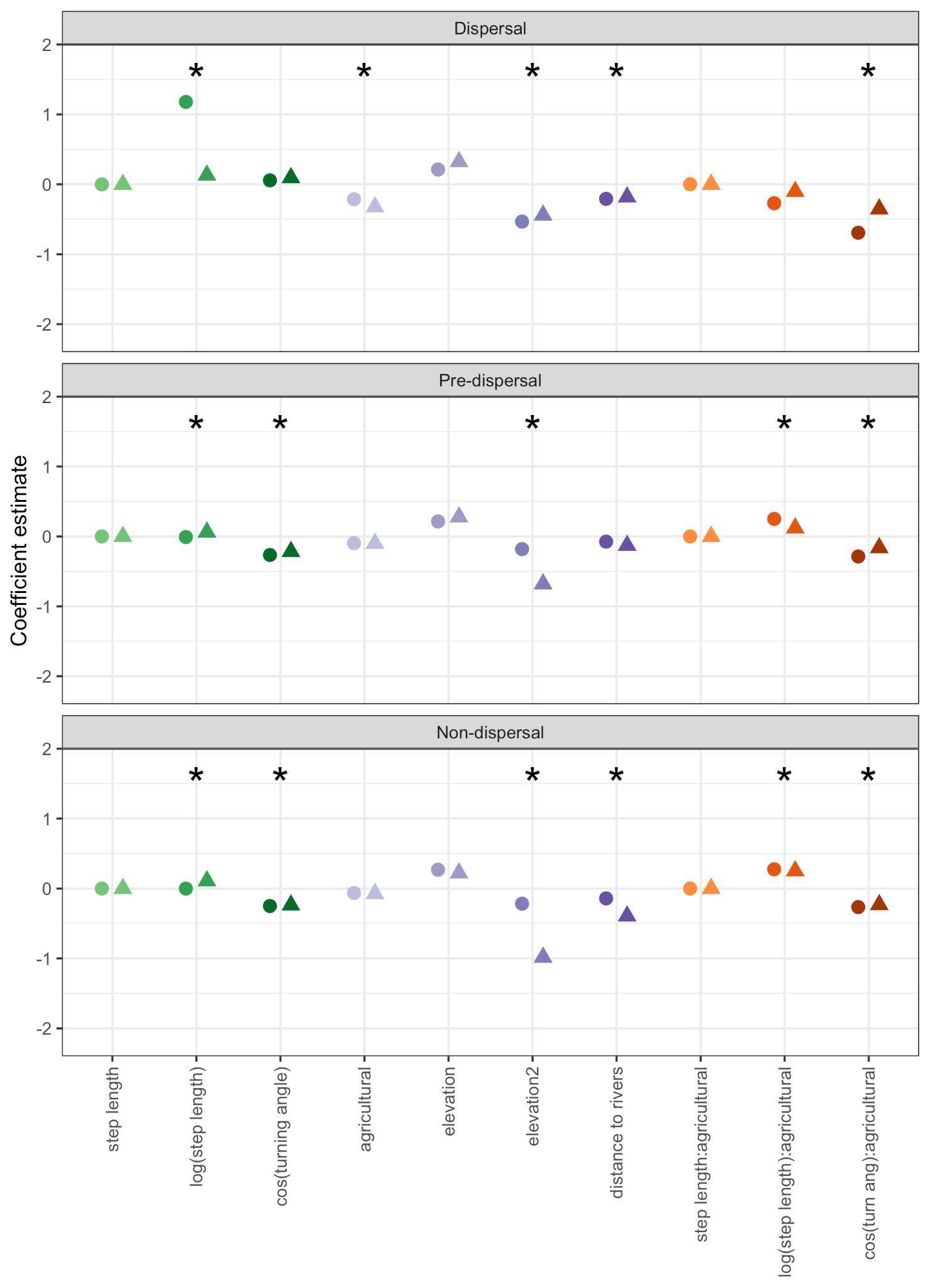


**Figure S16:** For juvenile male white-tailed deer in southwest Wisconsin, comparisons of coefficient estimates (un-exponentiated) from individual-level (circles) and population-level (triangles) integrated step selection functions (iSSFs) by movement state. Asterisks indicate variables that were statistically significant in population-level models. For simplicity, any *p*-value <0.05 is given by a single asterisk. Colors indicate type of variable, with main effect movement variables in green, main effect habitat variables in purple, and interactions between movement and habitat variables in orange. Note that elevation and elevation2 variables correspond to the second order polynomial for elevation used in models.


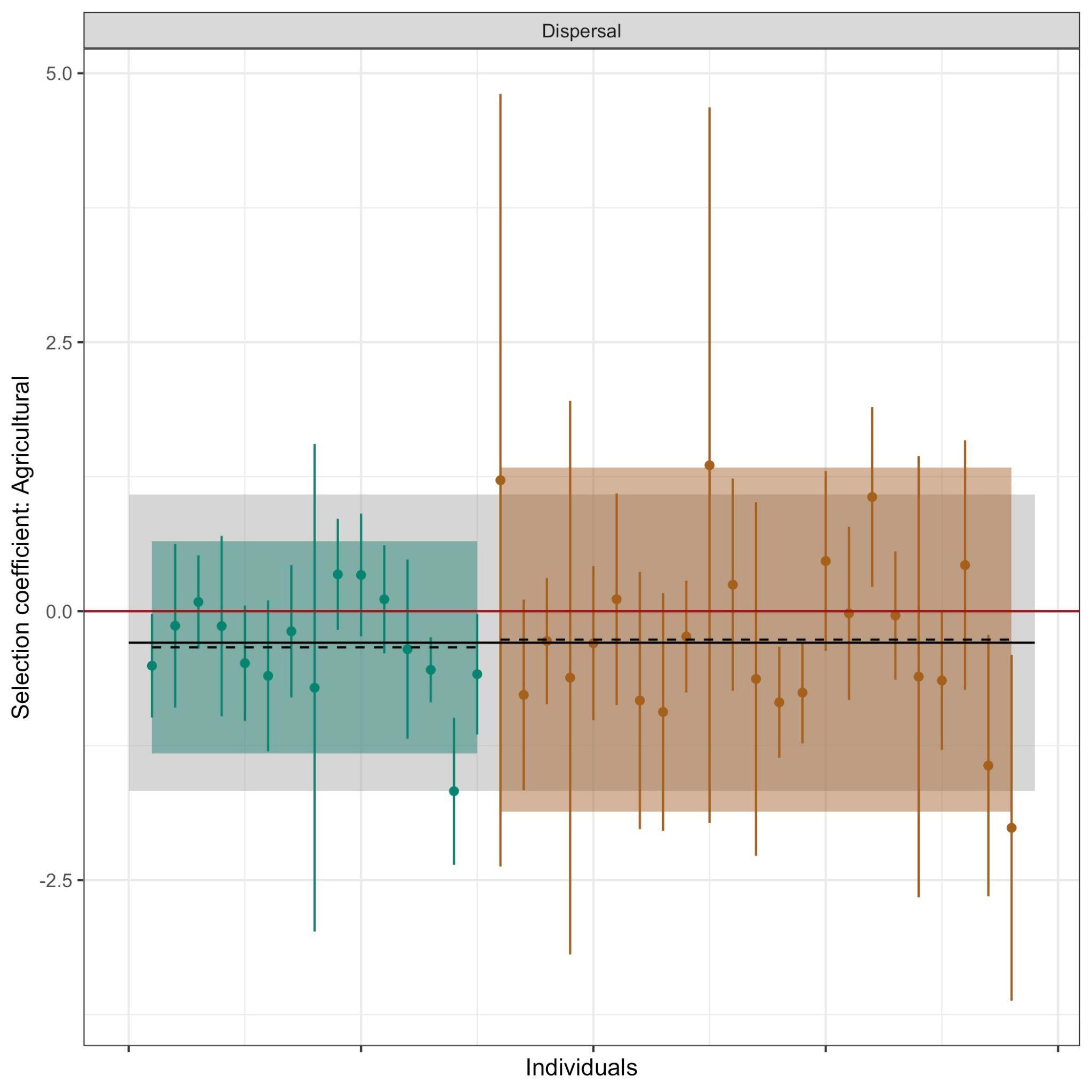


**Figure S17:** Main effect of agricultural land (un-exponentiated coefficient estimates) from individual-level step selection functions for southwest Wisconsin juvenile male white-tailed deer dispersal movements with locations recorded every one hour. Points with corresponding vertical lines show individual coefficient estimates and 95% confidence intervals. Colors correspond to seasons, with spring results given in green (left) and fall results in brown (right). The solid black line and background gray box show the mean coefficient estimate and 95% confidence interval (respectively) for each movement state. Dashed lines and colored boxes show the mean coefficient estimate and 95% confidence interval (respectively) stratified by season. The red line highlights a selection coefficient of 0, which corresponds to no selection or avoidance. Note that one outlier individual was removed for this plot; this individual’s coefficient estimate was strongly negative, so removing this individual conservatively prevented skewing the median further negative.


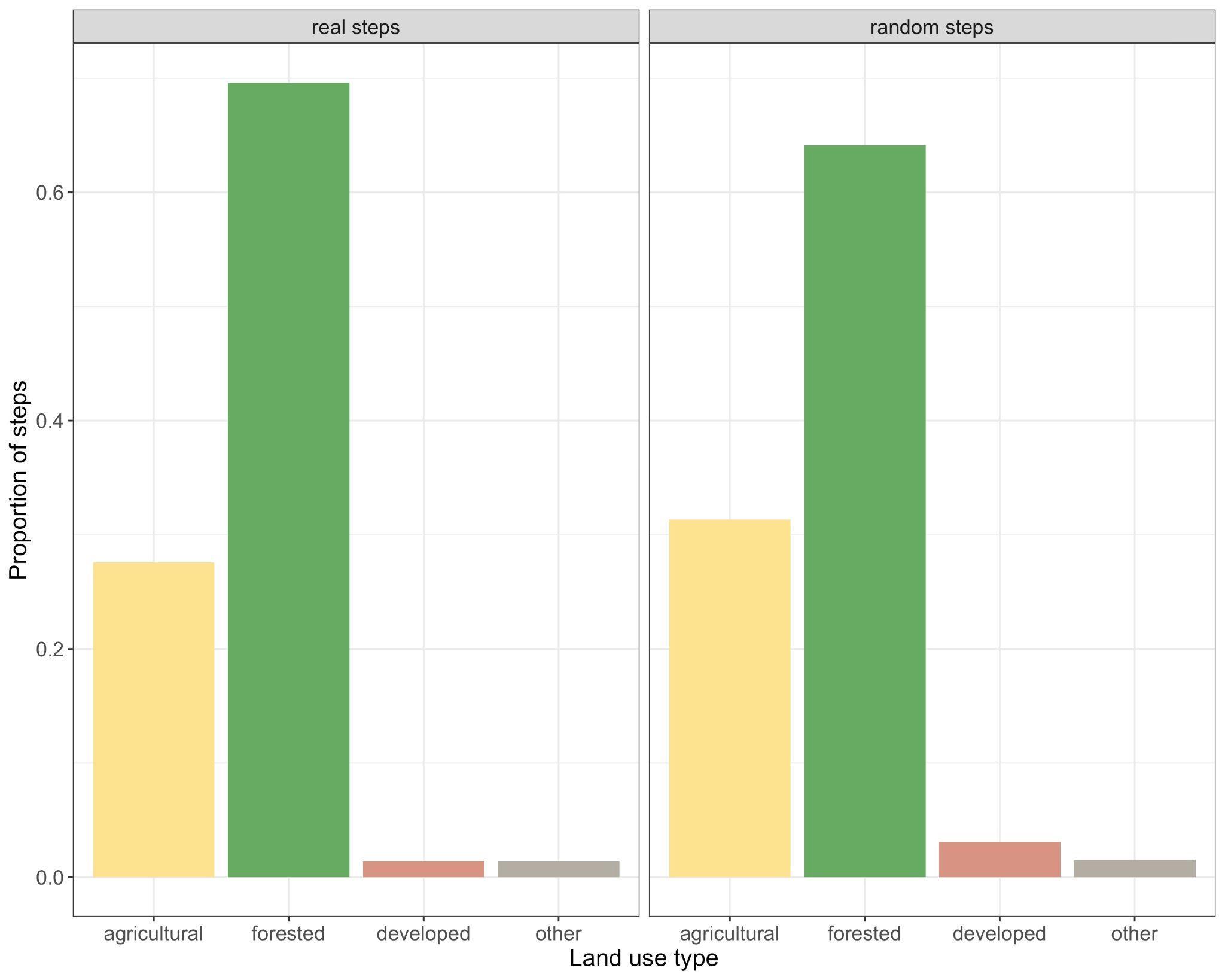


### Figure S18: Land use classifications of real (left) and random (right) steps for all 4-hourly movement trajectories that were used in the integrated step selection function (iSSF) analysis for southwest Wisconsin juvenile male white-tailed deer. Bars show proportions of steps per land use type, demonstrating that nearly all steps were in agricultural or forested land types. For real steps, the proportion of steps that were not in agricultural or forested land types (i.e., were in developed or other) was 2.8%; for random steps, 4.6%.

#

# SUPPLEMENTARY REFERENCES

1. Passoni G, Coulson T, Ranc N, Corradini A, Hewison AJM, Ciuti S, et al. Roads constrain movement across behavioural processes in a partially migratory ungulate. Mov Ecol. 2021;9:57.

2. Calabrese JM, Fleming CH, Gurarie E. Ctmm: An r package for analyzing animal relocation data as a continuous-time stochastic process. Methods Ecol Evol. 2016;7:1124–32.

3. Fleming CH, Fagan WF, Mueller T, Olson KA, Leimgruber P, Calabrese JM. Rigorous home range estimation with movement data: a new autocorrelated kernel density estimator. Ecology. 2015;96:1182–8.

4. Signer J, Fieberg J, Avgar T. Animal movement tools (amt): R package for managing tracking data and conducting habitat selection analyses. Ecol Evol. 2019;9:880–90.

5. Fieberg J, Signer J, Smith B, Avgar T. A “How to” guide for interpreting parameters in habitat-selection analyses. J Anim Ecol. 2021;90:1027–43.

6. Schnute JT, Groot K. Statistical analysis of animal orientation data. Anim Behav. 1992;43:15–33.

7. Fitak RR, Johnsen S. Bringing the analysis of animal orientation data full circle: model-based approaches with maximum likelihood. J Exp Biol. 2017;220:3878–82.
